# *ZMPSTE24* missense mutations that cause progeroid diseases decrease prelamin A cleavage activity and/or protein stability

**DOI:** 10.1242/dmm.033670

**Published:** 2018-07-13

**Authors:** Eric D. Spear, Erh-Ting Hsu, Laiyin Nie, Elisabeth P. Carpenter, Christine A. Hrycyna, Susan Michaelis

**Affiliations:** 1 Department of Cell Biology, The Johns Hopkins School of Medicine, Baltimore, MD 21205, USA; 2 Department of Chemistry, Purdue University, West Lafayette, IN 47907, USA; 3 Structural Genomics Consortium, University of Oxford, Oxford OX3 7DQ, UK

**Keywords:** Lamin A processing, *Saccharomyces cerevisiae*, Ubiquitin-proteasome system, Progeria disease

## Abstract

The human zinc metalloprotease ZMPSTE24 is an integral membrane protein crucial for the final step in the biogenesis of the nuclear scaffold protein lamin A, encoded by *LMNA*. After farnesylation and carboxyl methylation of its C-terminal CAAX motif, the lamin A precursor (prelamin A) undergoes proteolytic removal of its modified C-terminal 15 amino acids by ZMPSTE24. Mutations in *LMNA* or *ZMPSTE24* that impede this prelamin A cleavage step cause the premature aging disease Hutchinson-Gilford progeria syndrome (HGPS), and the related progeroid disorders mandibuloacral dysplasia type B (MAD-B) and restrictive dermopathy (RD). Here, we report the development of a ‘humanized yeast system’ to assay ZMPSTE24-dependent cleavage of prelamin A and examine the eight known disease-associated *ZMPSTE24* missense mutations. All mutations show diminished prelamin A processing and fall into three classes, with defects in activity, protein stability or both. Notably, some ZMPSTE24 mutants can be rescued by deleting the E3 ubiquitin ligase Doa10, involved in endoplasmic reticulum (ER)-associated degradation of misfolded membrane proteins, or by treatment with the proteasome inhibitor bortezomib. This finding may have important therapeutic implications for some patients. We also show that ZMPSTE24-mediated prelamin A cleavage can be uncoupled from the recently discovered role of ZMPSTE24 in clearance of ER membrane translocon-clogged substrates. Together with the crystal structure of ZMPSTE24, this humanized yeast system can guide structure-function studies to uncover mechanisms of prelamin A cleavage, translocon unclogging, and membrane protein folding and stability.

## Abstract (precis)

**Summary:** The zinc metalloprotease ZMPSTE24 performs the final step of prelamin A processing. Here, a yeast-based system shows differences in protein stability and activity for alleles of *ZMPSTE24* that cause progeria disease.

## INTRODUCTION

The integral membrane zinc metalloprotease ZMPSTE24 has a crucial role in human health and longevity through its role in the maturation of the nuclear scaffold protein lamin A from its precursor, prelamin A (Bergo et al., 2002; Michaelis and Hrycyna, 2013; Pendas et al., 2002). Mature lamin A, together with nuclear lamins B and C, contributes to the structural integrity and proper functioning of the nucleus (Butin-Israeli et al., 2012; Dechat et al., 2010; Dittmer and Misteli, 2011; Dorado and Andrés, 2017; Gerace and Huber, 2012; Gruenbaum and Foisner, 2015; Mattout et al., 2006). Defects in prelamin A processing by ZMPSTE24 are a primary cause of progeria (Capell and Collins, 2006; Davies et al., 2009; Gordon et al., 2014; Michaelis and Hrycyna, 2013). The premature aging disorder Hutchinson-Gilford progeria syndrome (HGPS; OMIM #176670) results from mutations in the *LMNA* gene (encoding prelamin A) that block ZMPSTE24 processing, whereas the related progeroid diseases mandibuloacral dysplasia type B (MAD-B; OMIM #608612) and restrictive dermopathy (RD; OMIM #275210) result from mutations in *ZMPSTE24* that diminish protease function (Barrowman et al., 2012b; Davies et al., 2009; De Sandre-Giovannoli et al., 2003; Eriksson et al., 2003; Navarro et al., 2014). Understanding the mechanistic details of prelamin A processing by ZMPSTE24 is thus crucial for designing therapeutic approaches for these progeroid diseases and might also provide insights into the normal physiological aging process.

**Fig. 1.**
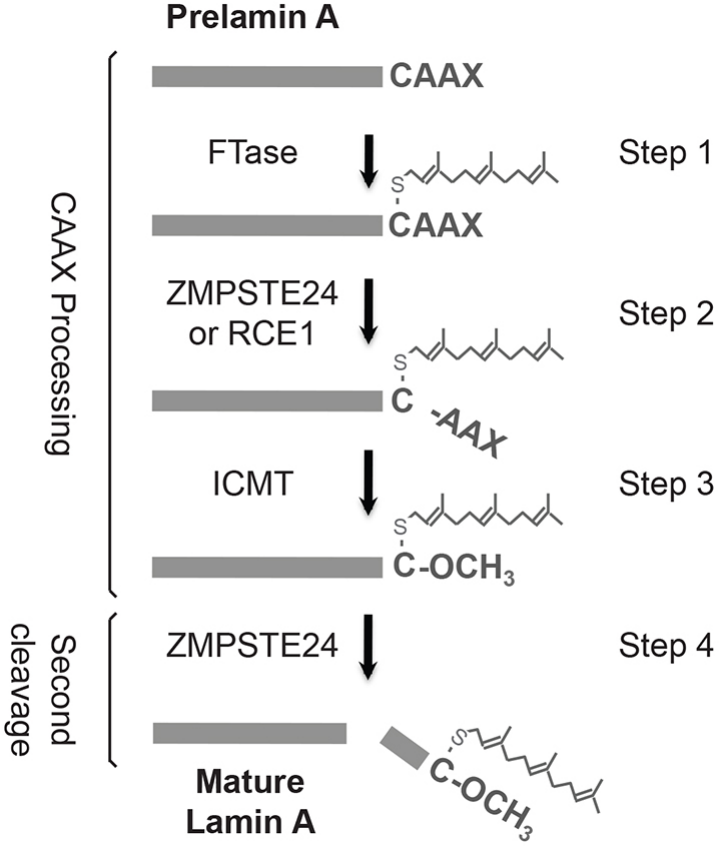
**The prelamin A biogenesis pathway.** The four steps of prelamin A post-translational processing shown here are described in the text. The lipid farnesyl (a 15-carbon-long isoprenoid lipid) and the carboxyl methyl group (O-CH_3_) are indicated. The enzymes that mediate CAAX processing are shown: farnesyltransferase (FTase), the proteases ZMPSTE24 and Ras-converting enzyme (RCE1) and the isoprenylcysteine carboxylmethyl transferase (ICMT). It should be noted that although step 2 in CAAX processing can be carried out redundantly for prelamin A either by ZMPSTE24 or RCE1, step 4 of prelamin A processing is solely mediated by ZMPSTE24. When ZMPSTE24 is absent, processing is blocked at step 4 and not step 2, as RCE1 is present (Varela et al., 2008; C.A.H., E.-T.H. and S.M., unpublished data).

The post-translational maturation of prelamin A is a multistep process. Prelamin A contains a C-terminal CAAX motif (where C is cysteine, A is usually an aliphatic amino acid and X is any residue). Like other CAAX proteins, prelamin A undergoes a series of three reactions, referred to as CAAX processing (Fig. 1; steps 1-3), which includes farnesylation of cysteine, proteolytic removal of the AAX residues mediated redundantly by ZMPSTE24 or Ras-converting enzyme 1 (RCE1), and carboxyl methylation of the farnesylated cysteine by isoprenylcysteine methyltransferase (ICMT) (Davies et al., 2009; Michaelis and Barrowman, 2012; Michaelis and Hrycyna, 2013; Wang and Casey, 2016). Prelamin A is distinct from all other CAAX proteins in higher eukaryotes in that, following CAAX processing, prelamin A undergoes a second endoproteolytic cleavage event uniquely mediated by ZMPSTE24 (Fig. 1; step 4). This second cleavage removes the C-terminal 15 amino acids, including the modified cysteine residue, to yield mature lamin A (Bergo et al., 2002; Pendas et al., 2002). In progeroid disorders, this second ZMPSTE24-promoted cleavage of prelamin A is compromised, leading to the accumulation of a permanently farnesylated and carboxyl methylated form of prelamin A, which is the toxic ‘culprit’ in these diseases (Davies et al., 2009; Gordon et al., 2014; Worman et al., 2009).

MAD-B, HGPS and RD represent a spectrum of disorders of increasing severity (Barrowman et al., 2012b; Navarro et al., 2014). In HGPS, the best studied of these disorders, children manifest accelerated aging symptoms starting at one year of age, including failure to thrive, lipodystrophy, hair loss, joint ailments and cardiovascular disease, and they typically die in their mid-teens from heart attack or stroke. Nearly all HGPS patients harbor a dominant *LMNA* mutation that, through altered splicing, generates an internally deleted version of prelamin A called progerin, which retains its CAAX motif but lacks the ZMPSTE24 cleavage site and causes disease phenotypes (De Sandre-Giovannoli et al., 2003; Eriksson et al., 2003; Gordon et al., 2014; Merideth et al., 2008). The disorders RD and MAD-B are a consequence of recessive mutations in *ZMPSTE24*, and result from the accumulation of full-length prelamin A that is permanently farnesylated and carboxyl methylated. RD is far more severe than HGPS, being fatal at or before birth, and is due to complete loss of ZMPSTE24 function resulting from null mutations (frameshifts, premature termination or large deletions) in both copies of *ZMPSTE24* (Moulson et al., 2005; Navarro et al., 2005, 2014; Smigiel et al., 2010). By contrast, MAD-B is generally milder than HGPS: patients have variable survival rates and disease severity, yet all exhibit lipodystrophy as a major disease phenotype. Individuals with MAD-B have one *ZMPSTE24* null allele and one *ZMPSTE24* missense allele that provides reduced but residual function (Table 1) (Agarwal et al., 2003; Barrowman et al., 2012b; Navarro et al., 2014). In general, the severity of these three progeroid diseases reflects the amount of permanently farnesylated and carboxyl methylated prelamin A that accumulates per cell. Recently, individuals with metabolic syndrome and non-alcoholic fatty liver disease (NAFLD), disorders both associated with lipodystrophy, were also found to have a *ZMPSTE24* missense mutation (Table 1) (Brady et al., 2018; Dutour et al., 2011; Galant et al., 2016). In addition, diminished ZMPSTE24 processing of prelamin A might be important in normal aging, as suggested by a study showing that prelamin A accumulation occurs in blood vessels from aging, and not young, individuals (Ragnauth et al., 2010). Because of the importance of diminished ZMPSTE24 processing of prelamin A in progeroid disease and possibly during normal aging, understanding the detailed mechanism of ZMPSTE24 is a key area of research.

**Table 1.**
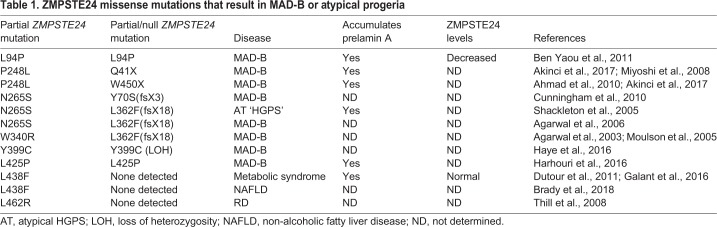
**ZMPSTE24 missense mutations that result in MAD-B or atypical progeria**

ZMPSTE24 is widely conserved in eukaryotes ranging from yeast to mammals (Barrowman and Michaelis, 2009; Pryor et al., 2013; Quigley et al., 2013). The *Saccharomyces cerevisiae* homolog Ste24 is the founding member of this family and was discovered on the basis of its role in the proteolytic maturation of the secreted yeast mating pheromone a-factor (Barrowman and Michaelis, 2011; Boyartchuk and Rine, 1998; Fujimura-Kamada et al., 1997; Michaelis and Barrowman, 2012; Tam et al., 1998). Prelamin A and the a-factor precursor are distinct from other CAAX proteins, as they are the only ones that undergo additional cleavage by ZMPSTE24/Ste24 after CAAX processing is completed (Barrowman and Michaelis, 2011; Beck et al., 1990; Bergo et al., 2002; Pendas et al., 2002; Sinensky et al., 1994). ZMPSTE24 and its homologs contain seven transmembrane spans and a consensus zinc metalloprotease HEXXH motif (where H is histidine, E is glutamate and X is any amino acid), which is crucial for coordinating zinc and performing catalysis (Barrowman et al., 2012b; Quigley et al., 2013). The recently solved X-ray crystal structure of human ZMPSTE24, and that of the virtually superimposable yeast Ste24, show it to represent a completely novel class of protease (Clark et al., 2017; Pryor et al., 2013; Quigley et al., 2013). The seven helical spans of ZMPSTE24 form a voluminous intramembrane ‘hollow’ chamber; the HEXXH catalytic domain is positioned such that it faces the interior of the chamber with a side portal(s) in the chamber presumably providing a site for prelamin A entry. This unusual ZMPSTE24 structure raises important functional questions, including how ZMPSTE24 mediates specificity for prelamin A access into its chamber, what residues are involved in positioning prelamin A for its cleavage(s), and what might be the role of the large chamber of ZMPSTE24. The answers to these questions are of fundamental biological interest and will help us to understand how specific ZMPSTE24 disease alleles malfunction and might be corrected. This information might also shed light on how certain HIV protease inhibitors, such as lopinavir, are able to inhibit ZMPSTE24 (Coffinier et al., 2007; Mehmood et al., 2016). Such insights could also have relevance to physiological aging.

ZMPSTE24 is dually localized in the inner nuclear and endoplasmic reticulum (ER) membranes (Barrowman et al., 2008) and performs cellular functions in addition to its well-established role in the proteolytic maturation of prelamin A and a-factor. Recent work shows that ZMPSTE24 has a role in protein quality by clearing ‘clogged’ Sec61 translocons of post-translationally secreted proteins that have aberrantly folded while in the process of translocation (Ast et al., 2016; Kayatekin et al., 2018). Intriguingly, a role for ZMPSTE24 in defending cells against a wide variety of enveloped viruses, independent of its catalytic activity, has also been recently reported (Fu et al., 2017).

Because of the importance of ZMPSTE24 in human health and disease and its novel structure, it would be advantageous to have a high-throughput system to probe structure-function relationships in this protease. Here, we report a ‘humanized yeast system’ to specifically assay the second ZMPSTE24 cleavage step in prelamin A maturation (Fig. 1; step 4). We show that the eight currently known disease-causing ZMPSTE24 missense alleles (Table 1) all have decreased prelamin A cleavage *in vivo* and fall into distinct classes: those that only affect cleavage activity, those that affect *in vivo* protein stability through ER-associated degradation (ERAD) by the ubiquitin-proteasome system (UPS), and those that affect both. Notably, for two unstable ZMPSTE24 disease mutants, P248L and W340R, when ubiquitylation or proteasome activity is blocked, both their stability and catalytic activity are significantly restored. These findings have implications for therapeutic strategies that could ultimately optimize ‘personalized medicine’ approaches. The *in vivo* assay system we present here, along with the ease of gene manipulation and genetic strategies available in yeast, hold promise for future high-throughput structure-function studies on ZMPSTE24.

## RESULTS

### ZMPSTE24 can perform the upstream cleavage of its *bona fide* substrate prelamin A in yeast

We previously showed that human ZMPSTE24 could functionally replace its yeast homolog Ste24 for the proteolytic maturation of its non-native substrate, the yeast mating pheromone a-factor (Barrowman and Michaelis, 2009; Schmidt et al., 2000; Tam et al., 1998). We also developed an assay in which the extent of yeast mating broadly correlated with the severity of ZMPSTE24 disease alleles, such that those that cause RD (null alleles) show more severe mating defects than those that cause the milder disease MAD-B (missense alleles) (Barrowman et al., 2012b). However, the mating assay is less than ideal for the mechanistic dissection of ZMPSTE24, as it relies on ZMPSTE24-dependent cleavage of the cross-species substrate a-factor and because it cannot distinguish between the two cleavage activities. Because the unique step in prelamin A cleavage by ZMPSTE24 is the second cleavage, and because it is the lack of this step that causes progeroid diseases, we set out to develop a system to specifically measure this ZMPSTE24-mediated processing step for its *bona fide* substrate prelamin A, which is not normally present in *S. cerevisiae*.

**Fig. 2.**
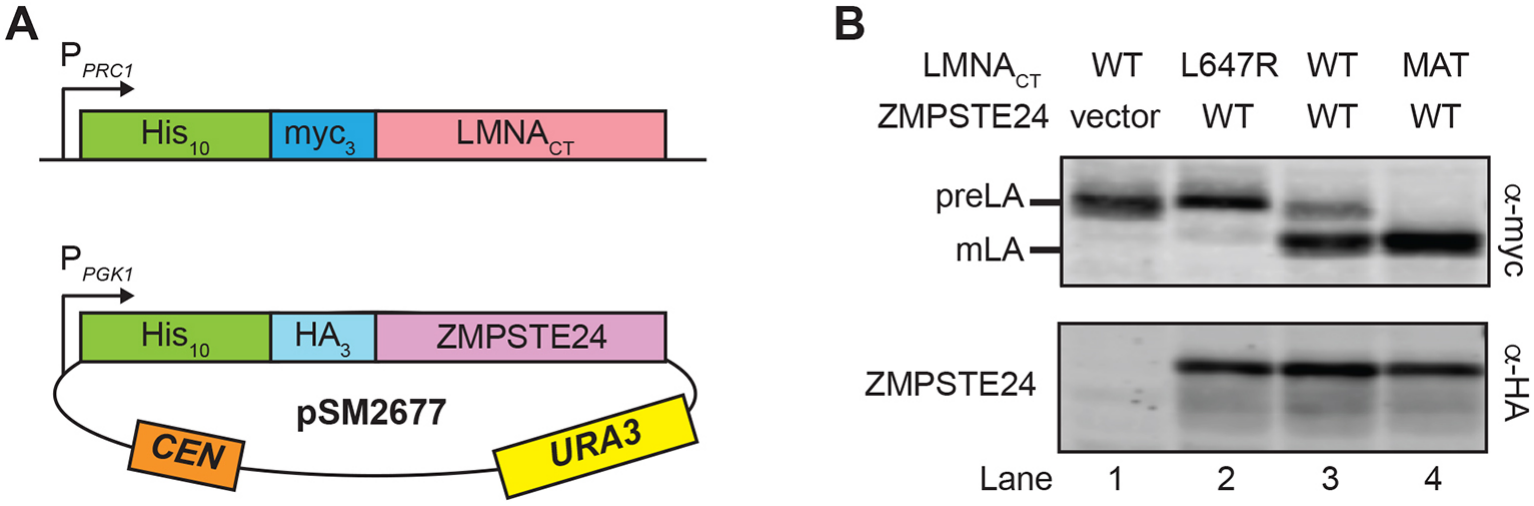
**Prelamin A is processed to mature lamin A by human ZMPSTE24 in yeast.** (A) Schematic of the humanized yeast system. The prelamin A model substrate contains amino acids 431-664 from the C terminus of human *LMNA* (referred to as LMNA_CT_) fused to a 10His-3myc epitope tag. The substrate is expressed from the *PRC1* promoter (P*_PRC1_*) and is chromosomally integrated into a *ste24*Δ strain background, resulting in strain SM6158. Full-length human ZMPSTE24 with an N-terminal 10His-3HA epitope tag is expressed from the *PGK1* promoter (P*_PGK1_*) on a *CEN URA3* plasmid (pSM2677; Barrowman et al., 2012b). (B) Lysates from *ste24*Δ strains expressing wild-type (WT) prelamin A (lanes 1 and 3), uncleavable prelamin A (lane 2, L647R) or mature lamin A (lane 4, MAT) and human ZMPSTE24 (lanes 2, 3 and 4) or vector alone (lane 1) were analyzed for prelamin A processing by SDS-PAGE and western blotting. Prelamin A (preLA) and mature lamin A (mLA) were detected with anti-myc antibodies; ZMPSTE24 was detected with anti-HA antibodies. Strains in lanes 1-4 are SM6158/pRS316, SM6177/pSM2677, SM6158/pSM2677 and SM6178/pSM2677, respectively.

To create a humanized yeast system to study ZMPSTE24-dependent processing of prelamin A, we expressed a C-terminal segment from the human prelamin A protein (amino acids 431-664) (Fig. 2A), which contains all the necessary signals for CAAX processing and the ZMPSTE24-dependent unique cleavage (Barrowman et al., 2008, 2012a). To serve as size markers for comparison, we also constructed a mutant prelamin A (L647R), which is known to be uncleavable by ZMPSTE24 in mammalian cells (Barrowman et al., 2012a; Hennekes and Nigg, 1994; Mallampalli et al., 2005; Wang et al., 2016), as well as a version expressing the correctly processed mature form of prelamin A (amino acids 431-646). All versions were tagged at the N terminus with 10His-3myc to allow detection by western blotting and were integrated into the yeast genome. In this humanized yeast system, ZMPSTE24 is expressed from a low-copy number yeast CEN plasmid and is tagged at the N terminus with 10His-3HA (Fig. 2A). Prelamin A cleavage is measured by quantitation of the mature and prelamin A species present in cells at steady state. Importantly, our strain background retains Rce1 to allow efficient removal of the AAX sequence (Fig. 1; step 2), thus eliminating any effect that mutant ZMPSTE24 proteins might have on this first cleavage step.

We first tested whether human ZMPSTE24 could process prelamin A in a *ste24*Δ strain. Plasmid-borne ZMPSTE24, but not vector alone, resulted in two bands observed by western blotting with anti-myc antibodies (Fig. 2B; top panel, compare lanes 1 and 3). Importantly, these bands co-migrated with the ‘uncleavable’ and ‘mature’ forms (Fig. 2B; top panel, lanes 2 and 4, respectively), suggesting that the prelamin A substrate was properly cleaved by ZMPSTE24. We note that yeast Ste24 can also cleave prelamin A (Fig. S1) and that prelamin A processing to the mature form by ZMPSTE24 can be enhanced by the addition of a second copy of ZMPSTE24 (Fig. S2).

**Fig. 3.**
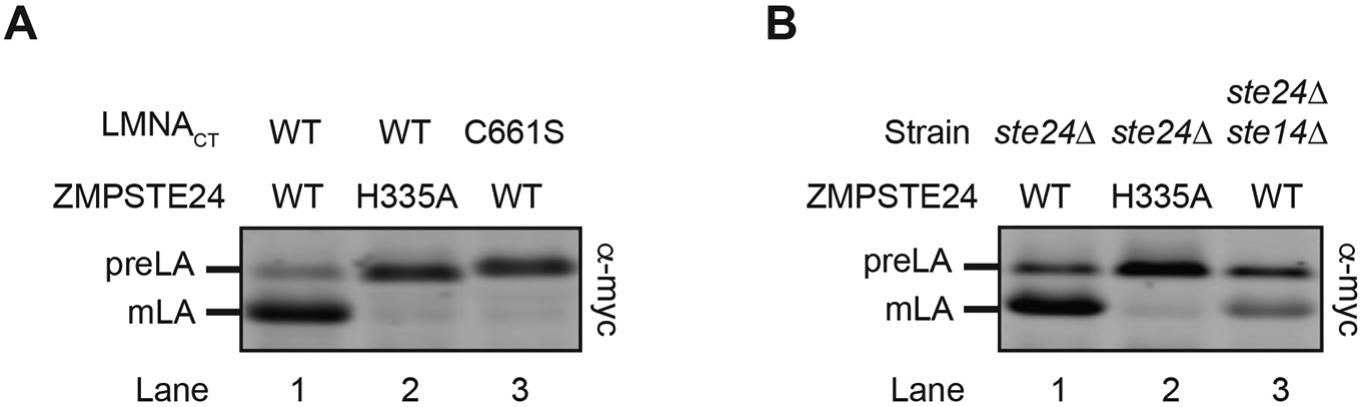
**Cleavage of prelamin A in yeast, as in mammalian cells, requires farnesylation of the CAAX motif and is diminished when carboxyl methylation is lacking.** (A) Prelamin A processing is blocked when farnesylation is absent. Prelamin A processing in *ste24*Δ strains expressing the indicated *LMNA*_CT_ (wild type or C661S) and *ZMPSTE24* (wild type and H335A) alleles was analyzed by SDS-PAGE and western blotting, as in Fig. 2. (B) The efficiency of prelamin A processing is reduced in a *ste14*Δ mutant strain. Prelamin A processing in strains expressing the indicated *ZMPSTE24* alleles was analyzed. Strains are *ste24*Δ only (lanes 1 and 2) or a *ste24*Δ*ste14*Δ double mutant (lane 3). Strains in lanes 1-3 are SM6158/pSM2677, SM6158/pSM2673 and SM6187/pSM2677, respectively. preLA, prelamin A; mLA, mature lamin A; WT, wild type.

We also tested whether prelamin A cleavage in yeast required the CAAX modifications farnesylation and carboxyl methylation, as it does in mammalian cells (Barrowman et al., 2008, 2012a). Wild-type ZMPSTE24, but not a catalytically dead mutant (H335A) resulted in mostly mature lamin A (Fig. 3A; compare lanes 1 and 2). Mutation of the CAAX motif cysteine to a serine (C661S), which prevents its farnesylation, completely blocked ZMPSTE24-dependent cleavage of prelamin A (Fig. 3A; lane 3). The unmodified C661S prelamin A migrated slightly more slowly than farnesylated prelamin A in the H335A ZMPSTE24 mutant (Fig. 3A; compare lanes 2 and 3), as has been previously observed (Yang et al., 2008). We also examined prelamin A cleavage in a *ste14*Δ strain, which lacks the yeast ICMT. As observed in mammalian cells (Barrowman et al., 2008, 2012a; Young et al., 2005), blocking carboxyl methylation of the prelamin A substrate has a modest, but discernible, effect on prelamin A cleavage (Fig. 3B; compare lane 3 to lane 1). Taken together, these experiments demonstrate that processing of prelamin A in yeast follows the same rules as in mammalian cells. Thus, we can use our humanized yeast system to study ZMPSTE24-dependent processing of its *bona fide* substrate prelamin A.

### All ZMPSTE24 disease missense mutations show reduced prelamin A cleavage and some exhibit a low level of protein

**Fig. 4.**
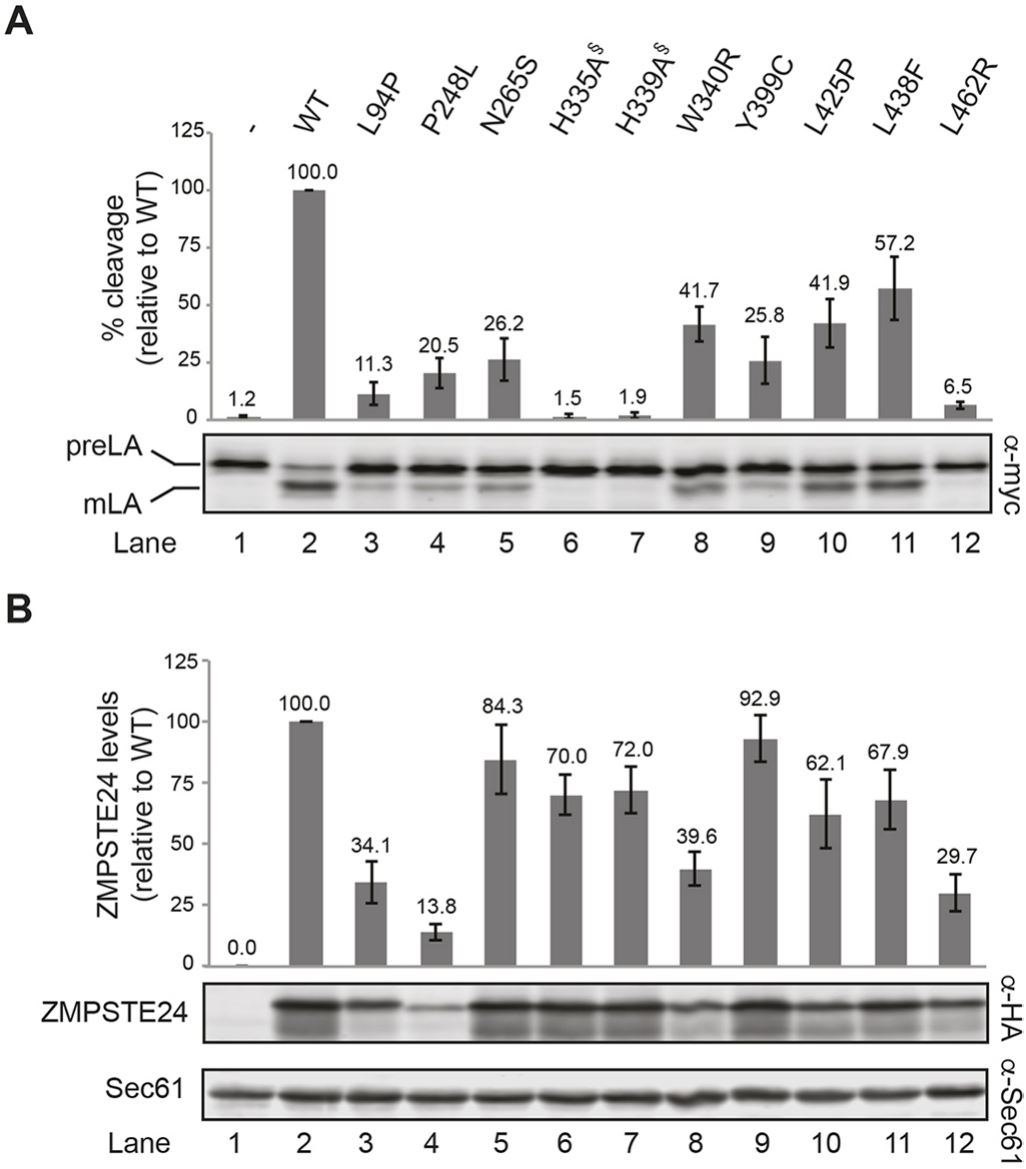
**ZMPSTE24 disease mutants show diminished prelamin A cleavage and for some alleles dramatically decreased protein levels.** Lysates from strain SM6158 (*ste24*Δ *myc-LMNA_CT_*) transformed with plasmids expressing the indicated HA-ZMPSTE24 alleles or vector only were analyzed by SDS-PAGE and western blotting. (A) Average (mean) percentage of prelamin A cleavage for each ZMPSTE24 variant was calculated from four independent experiments, with s.d. shown as error bars. For comparison, wild-type ZMPSTE24 cleavage was set to 100%; *P*<0.005 for all mutants compared with wild type. ^§^Catalytically dead mutants. (B) ZMPSTE24 proteins were detected with α-HA antibodies and the ZMPSTE24 levels were normalized to the loading control Sec61, with wild-type ZMPSTE24 set to 100%. The average (mean) and s.d. are shown for the same four experiments as in (A). *P*<0.05 for all mutants compared with wild type, except N265S and Y399C, which were not considered to be significantly different from wild type. We note that the multiple banding pattern seen here for ZMPSTE24 occurs not only in our yeast system, but also for endogenous ZMPSTE24 in mammalian cells (Pendas et al., 2002) and when ZMPSTE24 is expressed in other heterologous expression systems (Clark et al., 2017; E.P.C. and L.N., unpublished observations). The different mobilities might simply reflect distinct SDS-binding patterns for this protein or an, as yet, unknown modification. preLA, prelamin A; mLA, mature lamin A; WT, wild type.

One goal of developing a yeast *in vivo* cleavage assay was to determine whether particular *ZMPSTE24* disease alleles resulted in defective prelamin A cleavage, and by what mechanism(s), which ultimately might suggest therapeutic possibilities. Currently, eight different ZMPSTE24 substitution mutations are known to cause progeroid disorders (Table 1). We examined the processing efficiency of these alleles compared with wild-type ZMPSTE24. Also included in our panel are two mutations, H335A and H339A, known to abolish ZMPSTE24 activity by disrupting the zinc metalloprotease domain (H_335_EXXH_339_) (Barrowman and Michaelis, 2009; Barrowman et al., 2012b; Fujimura-Kamada et al., 1997; Quigley et al., 2013). As evident in Fig. 4A and summarized in Table 2, all of the mutations we examined showed reduced *in vivo* prelamin A cleavage compared with wild-type ZMPSTE24, albeit to widely varying degrees. For instance, L438F shows the highest residual activity at 57.2% of wild-type ZMPSTE24, whereas L462R shows the least at 6.5% (Fig. 4A; compare lanes 11 and 12 with lane 2). Notably, none of the disease alleles are as severe as the two catalytically dead mutants H335A and H339A (Fig. 4A; lanes 6 and 7), which have <2% wild-type ZMPSTE24 activity. Some of the mutations in our panel were previously shown to accumulate prelamin A in patient cells (L94P, P248L, N265S, L425P and L438F) (Ben Yaou et al., 2011; Dutour et al., 2011; Harhouri et al., 2016; Miyoshi et al., 2008; Shackleton et al., 2005), but directly comparing the extent of severity for these *ZMPSTE24* alleles was not possible in non-isogenic patient cells. The yeast system, however, is ideal for this purpose. Three of the mutants, W340R, Y399C and L462R, had never been examined for prelamin A processing defects. Thus, our yeast system has for the first time confirmed a potential molecular basis underlying these latter mutants (prelamin A accumulation) and allows us to compare levels of residual processing between all known ZMPSTE24 disease alleles.

**Table 2.**
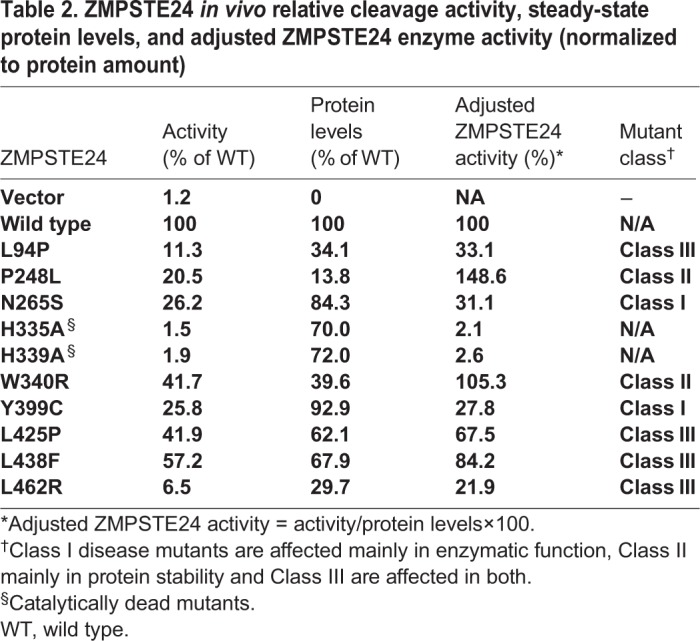
**ZMPSTE24 *in vivo* relative cleavage activity, steady-state protein levels, and adjusted ZMPSTE24 enzyme activity (normalized to protein amount)**

Given the non-conservative amino acid substitutions in several of the ZMPSTE24 mutants, we considered the possibility that decreased prelamin A cleavage could, at least in part, be the result of ZMPSTE24 misfolding and subsequent degradation (Fig. 4B). Indeed, western blotting revealed varying amounts of ZMPSTE24 for some mutants. It should be noted that ZMPSTE24 resolves as a major band with a faster migrating smearing pattern and a minor band below it, as observed previously (Clark et al., 2017). Such anomalous SDS-PAGE migration patterns are not uncommon for membrane proteins and reflect the unusual detergent-binding properties of their helical spans (Newman et al., 1981; Rath et al., 2009). Four of the mutants (L94P, P248L, W340R and L462R) showed steady-state ZMPSTE24 levels significantly less (<40%) than that of wild-type ZMPSTE24 (Fig. 4B; compare lanes 3, 4, 8 and 12 with lane 2). Notably, when prelamin A processing is normalized for the amount of ZMPSTE24 protein, two of the mutants (P248L and W340R) had an ‘adjusted ZMPSTE24 activity’ of 100% or higher (Table 2). This observation suggests that degradation, and not compromised catalytic activity, is the problem for these alleles (and in the section below, we find these two mutants are significantly active when their degradation is blocked). Strikingly, however, other mutants, including N265S and Y399C, displayed near-normal ZMPSTE24 protein levels, yet retained only ∼25-30% activity (Fig. 4A,B). Although less than wild type, these mutants still show far more activity than the catalytically dead mutants H335A and H339A (<2%) (Table 2).

Together, these results demonstrate that the humanized yeast system can differentiate three classes of ZMPSTE24 disease mutations: Class I mutants are those that affect mainly enzymatic activity (N265S and Y399C), Class II are those that affect mainly protein stability (P248L and W340R) and Class III are those that appear to affect both (L94P, L425P, L438F and L462R).

### Blocking ubiquitylation and degradation of some ZMPSTE24 disease mutants rescues the prelamin A cleavage defect

Mutations in transmembrane proteins like ZMPSTE24 can result in degradation by the UPS, causing disease despite the fact that their catalytic function remains intact. In some cases, enhancing the folding or blocking the degradation of these mutant proteins using pharmacological chaperones, proteasome inhibitors or mutants defective in ubiquitylation can rescue protein levels enough to restore function within the cell (Amaral, 2015; Brodsky, 2012; Guerriero and Brodsky, 2012). We therefore asked whether blocking the ubiquitylation and degradation of the most unstable ZMPSTE24 mutant proteins, L94P, P248L, W340R and L462R, could rescue prelamin A cleavage.

**Fig. 5.**
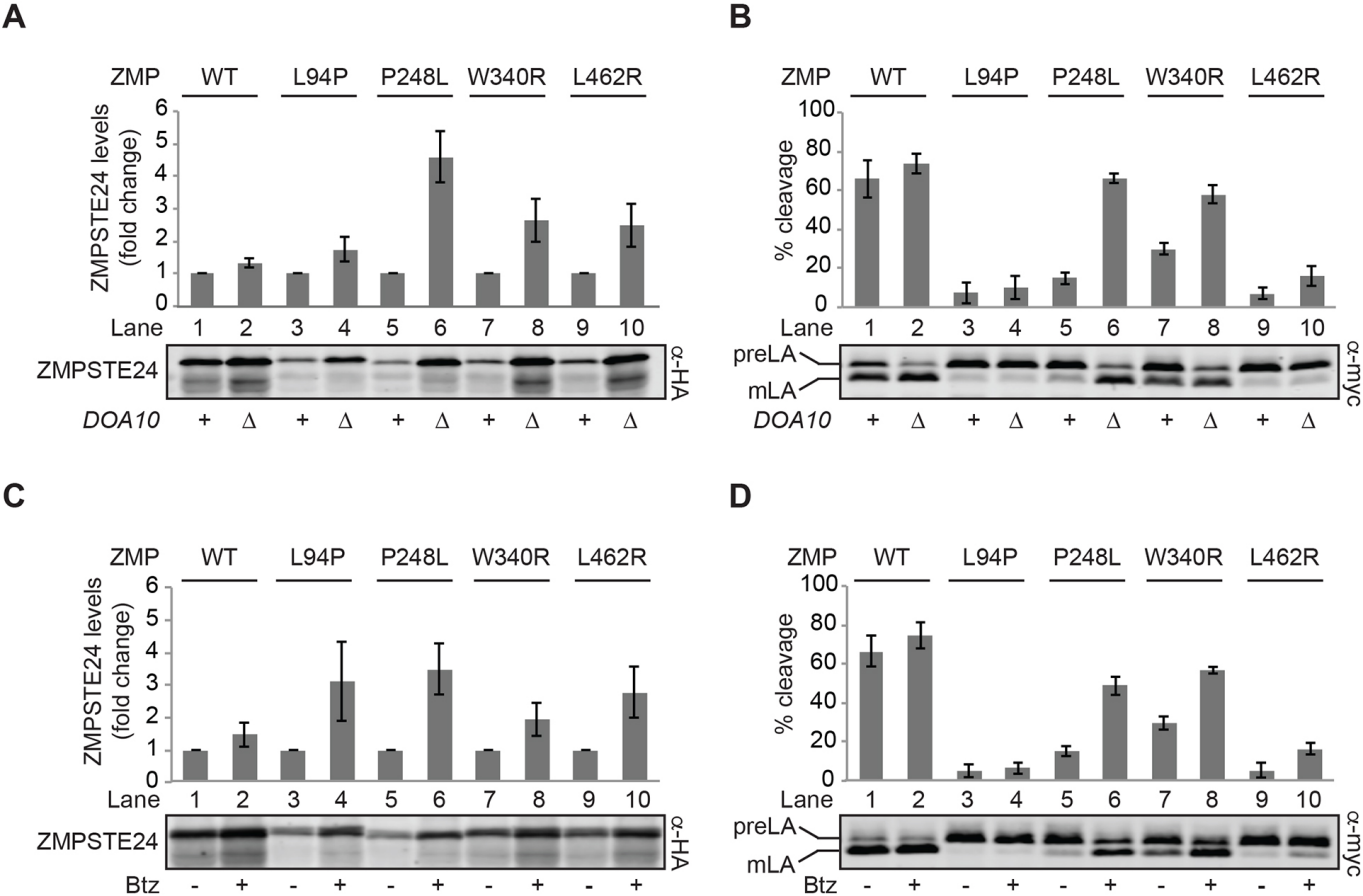
**Blocking the ubiquitin/proteasome-dependent degradation of mutant ZMPSTE24 proteins enhances prelamin A cleavage for some ZMPSTE24 disease variants.** (A,B) Examining the effects of blocking ubiquitylation. Strains SM6158 (*ste24*Δ *myc-LMNA_CT_*) or SM6184 (*ste24*Δ*doa10*Δ *myc-LMNA_CT_*) expressing the indicated ZMPSTE24 variants were analyzed by SDS-PAGE and western blotting using (A) α-HA and (B) α-myc antibodies. ZMPSTE24 protein levels were normalized against the loading control Sec61 (not shown). The *doa10*Δ mutant strain is designated as ‘Δ’ and the wild-type *DOA10* strain as ‘+’. Data shown is mean±s.d. for four independent experiments. *P*<0.05 for all comparisons between + and Δ for ZMPSTE24 protein levels; *P*<0.005 for P248L and W340R comparing activity (B). (C,D) Examining the effects of proteasome inhibition. To test the effect of proteasome inhibition on ZMPSTE24 protein levels and activity, strain SM6159 (*pdr5*Δ*ste24*Δ *myc-LMNA_CT_*) expressing the indicated ZMPSTE24 variants was treated with 20 µM bortezomib (+) or DMSO vehicle (−), as described in the Materials and Methods section. (C) HA-ZMPSTE24 proteins detected with anti-HA antibodies were normalized to the loading control Sec61 (not shown) and levels were expressed as the fold change between treated (+) and untreated (−) samples. A representative gel is shown, with the mean±s.d. for three independent experiments shown above; *P*<0.05 for all comparisons of mutant ZMPSTE24 proteins. (D) Prelamin A cleavage from the same samples shown in (C) was assessed with anti-myc antibodies; *P*<0.05 for P248L, W340R and L462R compared with wild type. preLA, prelamin A; mLA, mature lamin A; WT, wild type.

To test whether blocking ubiquitylation affected ZMPSTE24 protein levels and activity, we deleted the gene encoding Doa10, a dually localized ER and inner nuclear membrane E3 ligase known to ubiquitylate many misfolded transmembrane proteins and to target them for degradation (Deng and Hochstrasser, 2006; Huyer et al., 2004; Ravid et al., 2006; Swanson et al., 2001). Steady-state levels of the ZMPSTE24 mutant proteins increase ∼2-5-fold in the *doa10*Δ strain, indicating that all are substrates, either partially or fully, of this ubiquitin ligase (Fig. 5A; compare adjacent lanes for each disease mutant). Importantly, we observed that stabilization of P248L and W340R in the *doa10*Δ strain, but not of L94P or L462R, restored prelamin A cleavage activity to near wild-type levels (Fig. 5B; compare adjacent lanes 5 and 6, 7 and 8 versus 3 and 4, 9 and 10).

We also tested whether the proteasome inhibitor bortezomib had similar effects to the *doa10*Δ mutant. Treatment of cells with 20 µM bortezomib for 4 h resulted in ∼2-4-fold more protein for all ZMPSTE24 mutants compared with drug vehicle alone (Fig. 5C; compare – and + lanes for each variant). In agreement with the above results where ubiquitylation is blocked, both P248L and W340R showed enhanced prelamin A cleavage upon proteasome inhibition (Fig. 5D; compare lanes 5 and 6, and lanes 7 and 8). Notably, these same two mutants, P248L and W340R, had shown wild-type or better-adjusted ZMPSTE24 activity above (Table 2), when activity was normalized to the amount of ZMPSTE24 protein present. Taken together, these data suggest that some ZMPSTE24 patient mutations (Class II) are prematurely targeted for ubiquitin-mediated degradation, despite retaining catalytic activity. For patients with these alleles, therapeutic strategies that reverse the destruction of these otherwise functional enzymes by blocking their degradation could be beneficial.

### The prelamin A cleavage and Sec61 translocon clearance functions of ZMPSTE24 can be genetically separated

Recently, yeast Ste24 and mammalian ZMPSTE24 were shown to have a specialized role in controlling protein quality by handling post-translationally secreted proteins that prematurely fold while translocating across the ER membrane, thereby clogging the Sec61 translocation machinery (Ast et al., 2016). Clearance of a reporter ‘clogger’ protein by yeast Ste24 or heterologously expressed human ZMPSTE24 in *ste24*Δ yeast cells required their catalytic activity. Although four ZMPSTE24 disease alleles, including L94P, P248L, W340R and L438F, were previously assayed for their ability to clear the clogger substrate and shown to be defective to varying extents (Ast et al., 2016), four additional mutants in our current study were not tested.

**Fig. 6.**
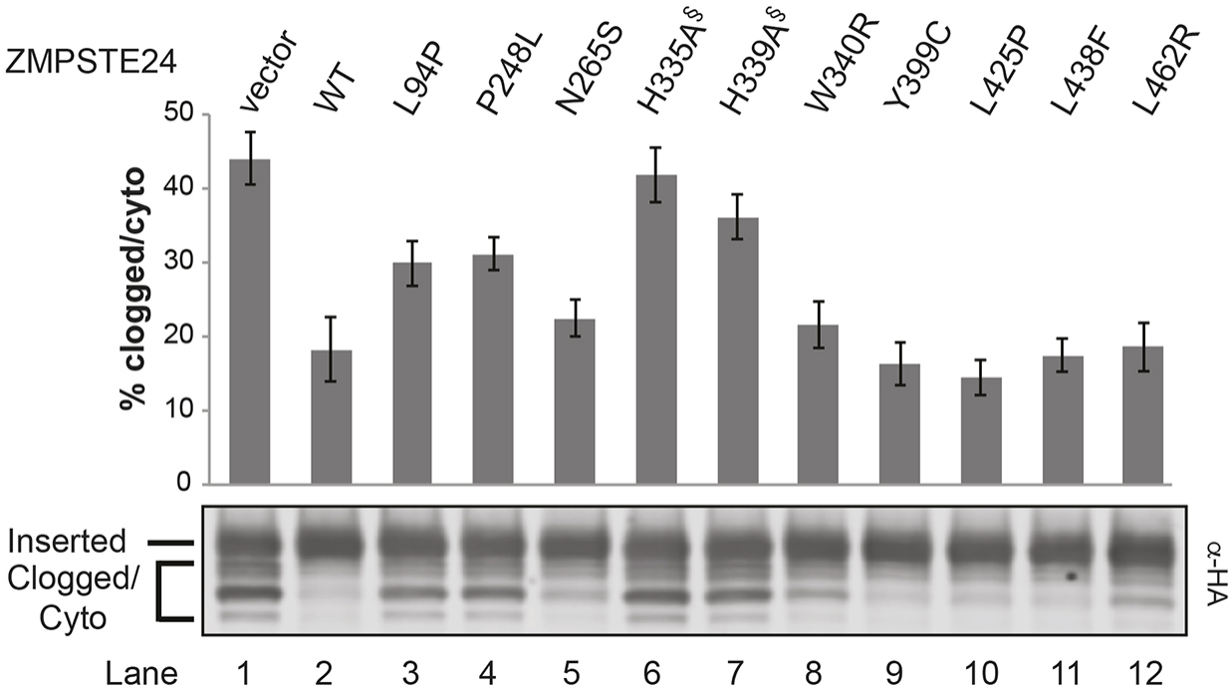
**Comparison of ZMPSTE24 mutants for clearance of the clogger protein.** Strain SM6117 (*ste24*Δ *P_GAL_-Clogger-HA*) transformed with the indicated ZMPSTE24 plasmids was induced to express the clogger protein by addition of galactose, as described in the Materials and Methods section. Lysates were resolved by SDS-PAGE and probed with α-HA antibodies to detect the clogger. The inserted and clogged or cytoplasmic species are indicated, with the percentage clogged/cytoplasmic indicated on the *y*-axis. Data shown are the mean±s.e.m. for five individual experiments; *P*<0.05 for vector, P248L, H335A and H339A compared with wild type (WT). ^§^Catalytically dead mutants.

The clogger reporter is a chimeric protein comprised of the yeast glycoprotein Pdi1 fused to the clogging element bacterial dihydrofolate reductase (DHFR), followed by a stretch of N-linked glycosylation sequences (Ast et al., 2016). Its complete translocation into the ER lumen is observed as an SDS-PAGE mobility shift when all N-glycosylation sites (in both Pdi1 and the C terminus) are modified. The hemi-glycosylated substrate is assumed to be partially translocated (clogged) and the unmodified protein represents a cytoplasmic pool that accumulates upon translocon clogging (Fig. 6). As observed previously, *ste24*Δ cells transformed with vector alone or the catalytically dead ZMPSTE24 variants H335A and H339A had the most severe defects (Fig. 6; lanes 1, 6 and 7, respectively) with 36-44% of the reporter accumulating in the ‘clogged/cytoplasmic’ forms, compared with only ∼18% for wild-type ZMPSTE24 (Fig. 6; lane 2). Likewise, L94P and P248L, which have severe prelamin A processing defects also showed significant clogged/cytoplasmic accumulation (∼30%) (Fig. 6; lanes 3 and 4). Surprisingly, however, other ZMPSTE24 mutants, including Y399C, L425P and L438F, despite showing prelamin A cleavage defects had little to no defects in clogger clearance (Fig. 6; lanes 5 and 9-11). L462R is also intriguing as it is relatively unstable and has a strong prelamin A cleavage defect, yet shows only a minor defect in clogger clearance (Fig. 6; lane 12). These findings suggest that although catalytic activity is required for both functions of ZMPSTE24, prelamin A processing and ‘declogging’ might differ in important ways mechanistically. For instance, mutant ZMPSTE24 proteins might be affected differently in their ability to be recruited to the Sec61 translocon or in their ability to permit access of the two different types of substrates (prelamin A versus clogged proteins) into their active-site chamber. It will be of interest to attempt to isolate ZMPSTE24 variants that can efficiently process prelamin A, but which are defective for clogger clearance.

## DISCUSSION

### A humanized yeast system for analysis of ZMPSTE24 cleavage of prelamin A

Gaining an understanding of how the structure of a protein dictates its function is facilitated by assaying the effect of specific mutations on activity, protein stability and interactions *in vivo*. For proteins involved in disease, this information can also provide invaluable insights into personalized medicine strategies, as exemplified by the customized therapies recently developed to treat cystic fibrosis patients with different disease alleles of the *CFTR* gene (Amaral, 2015). Here, we report the development of an *in vivo* humanized yeast system to determine the effect of *ZMPSTE24* disease mutations on the cleavage of prelamin A (Fig. 1; step 4), the step that is defective in progeroid diseases. Importantly, this yeast system allows us to measure both ZMPSTE24 prelamin A cleavage activity and *in vivo* protein stability, and has the potential to ultimately be scaled up for high-throughput analysis of a large number of mutant alleles. Our humanized yeast system retains all of the known requirements observed in mammalian cells (Figs 2 and 3) in that the prelamin A substrate must be farnesylated and carboxyl methylated for efficient cleavage. Moreover, mutation of a residue adjacent to the cleavage site in prelamin A (L647R) abolished processing in yeast, as it does in mammalian cells. Furthermore, mutation of the ZMPSTE24 catalytic motif HEHHX completely blocks prelamin A processing.

We previously used yeast to gain insight into *ZMPSTE24* disease alleles, based on the ability of human ZMPSTE24 to mediate the post-translational maturation of a non-native substrate, the yeast mating pheromone a-factor (Barrowman et al., 2012b). In that study, yeast mating efficiency was measured as a proxy for directly assessing substrate cleavage. We showed that RD null alleles were completely devoid of mating, whereas the five ZMPSTE24 MAD-B missense mutations tested all showed some residual mating activity. That study supported the notion that even a small amount of ZMPSTE24 function diminishes disease severity and is beneficial for patients. However, it was not possible to determine whether removal of the AAX sequence (Fig. 1; step 2), the final cleavage step (Fig. 1; step 4) or both were affected, as the *rce1*Δ*ste24*Δ strain used in that study required ZMPSTE24 for both of the a-factor processing events. In the current study, we have developed an improved and completely humanized yeast system (both substrate end enzyme are encoded by human genes). Because Rce1 is present to perform removal of the AAX sequence, the final prelamin A cleavage step is the reaction being measured. Our optimized system not only assays the ability of ZMPSTE24 to cleave its *bona fide* substrate human prelamin A, but also provides a good model for the conditions present in RD and MAD-B patient cells where ZMPSTE24 is mutated and RCE1 is present.

### ZMPSTE24 missense disease alleles show reduced prelamin A cleavage *in vivo* and define three mutant classes

We tested the eight currently known *ZMPSTE24* missense alleles implicated in progeroid diseases (Table 1), along with two catalytically dead alleles that alter the HEXXH domain (H335A and H339A). Although all of the disease mutants exhibit decreased overall prelamin A cleavage compared with wild-type *ZMPSTE24*, all show residual prelamin A cleavage activity significantly greater than the catalytically dead alleles (Fig. 4 and Table 2). The mutants vary greatly, however, in the extent of remaining activity (6-57% that of wild type). Importantly, four of the ZMPSTE24 mutations (L94P, P248L, W340R and L462R) showed marked decreases in ZMPSTE24 protein levels (14-40% of the wild-type level), suggesting these mutations cause misfolding and subsequent degradation; for the other mutants, protein stability was only minimally affected. Taking into account both their activity and stability we can divide ZMPSTE24 disease alleles into three classes (Table 2): Class I mutations affect mainly cleavage activity (N265S and Y399C), Class II affect mainly protein stability (P248L and W340R), and Class III mutants affect both (L94P, L425P, L438F and L462R). None of the mutant alleles we tested influence the ER/perinuclear membrane localization of ZMPSTE24-GFP (Fig. S3) or HA-ZMPSTE24 (data not shown).

For the highly unstable mutant proteins (L94P, P248L, W340R and L462R), the UPS is largely responsible for their degradation, as their ZMPSTE24 protein levels are significantly restored when cells are treated with the proteasome inhibitor bortezomib or when expressed in a *doa10*Δ strain (Fig. 5A,C). Similarly, steady-state levels of the other ZMPSTE24 disease alleles, including the least unstable variants N265S, Y399C, L425P and L438F, increased in the *doa10*Δ mutant, although none as dramatically as P248L (Fig. S4). Notably, for the P248L and W340R (Class II) mutants, but not for the L94P and L462R (Class III) mutants, prelamin A cleavage is dramatically restored to near wild-type levels in the *doa10*Δ strain or following proteasome inhibition using bortezomib (Fig. 5B,D). This finding confirms the conclusion that the enzyme function of these two Class II mutant proteins remains largely intact, as also indicated by the adjusted ZMPSTE24 activity column in Table 2, despite the presence of mutations that target them for degradation. Indeed, preliminary experiments using purified ZMPSTE24 proteins show that the P248L and W340R variants retain significant prelamin A cleavage activity *in vitro* (E.P.C. and L.N., unpublished observations). P248L was also suggested to retain partial activity in a previous study based on a-factor production (Miyoshi et al., 2008).

It is not uncommon for a mutation that impairs folding only slightly to result in avid protein clearance by the UPS, but for that protein to retain residual function if its degradation is inhibited (Gardner et al., 2005). Thus, the protein quality control system is sometimes more aggressive than it needs to be and offers the potential for proteasome inhibitor drugs to be used to ameliorate disease. In the future, it will be of great interest to determine whether ZMPSTE24 protein levels and prelamin A processing can be restored in P248L and W340R disease patient cells by genetic or chemical manipulations of the UPS. Such a finding could point the way to personalized medicine approaches that would differ between patients with Class II mutations and patients with Class I and III mutations that affect protein function. Similar personalized medicine approaches have been successful for cystic fibrosis, in which different drug treatments have been developed for patients with different classes of CFTR mutations (De Boeck and Amaral, 2016). Although not yet directly tested in clinical trials, all patients with mutated versions of ZMPSTE24 are predicted to benefit from farnesyltransferase inhibitors (FTIs), which should render full-length prelamin A unmodified and thus less harmul, as it does for progerin in HGPS (Gordon et al., 2014). Patients with Class II *ZMPSTE24* mutations, however, could potentially improve even more through use of a combination of FTIs and UPS inhibitors.

### Analysis of genetic dominance for L462R and L438F ZMPSTE24 alleles

Genetic pedigree analysis and patient genotypes indicate that *ZMPSTE24* mutations are generally recessive. Thus, disease is usually not manifested in individuals if one of their two *ZMPSTE24* alleles is wild type. However, two patient mutations, L462R and L438F, have been suggested to be exceptions and could be dominant, as mutations in the second *ZMPSTE24* allele were not identified in these patients by sequence analysis of exons (Table 1). These patients have RD and metabolic syndrome, respectively. Surprisingly, however, the healthy mother of the L462R RD patient shared the same apparent genotype (*ZMPSTE24^+/L462^*) as her affected offspring, arguing against dominance (Thill et al., 2008). We propose that L462R is actually recessive and that an, as yet, undetected mutation either inside (and missed, as has happened previously; Navarro et al., 2005) or outside the coding region (e.g. promoter, intron, etc.) might inactivate the second seemingly wild-type *ZMPSTE24* allele of the child with RD. A similar explanation could account for disease in L438F patients as well. Supporting this hypothesis and arguing against dominance, we found that an additional wild-type copy of *ZMPSTE24* could efficiently suppress the prelamin A cleavage defect observed in all *ZMPSTE24* disease alleles, including L462R and L438F (Fig. S5).

In light of the possibility that both L462R and L438F are actually recessive, an additional aspect of these mutations deserves mention. Most *ZMPSTE24* missense mutations cause MAD-B. It is notable, however, that we found L462R to be the most severe of the alleles studied here, whereas L438F is the least severe and shows significant residual activity (Fig. 4 and Table 2). This observation might explain why the L462R mutation leads to the disease RD, which is more severe than MAD-B, whereas L438F leads to metabolic disorder or NAFLD, diseases that are milder than MAD-B (Brady et al., 2018; Dutour et al., 2011; Galant et al., 2016).

### Some, but not all, disease alleles affect the ability of ZMPSTE24 to clear clogged translocons

Our studies suggest that the recently reported role of ZMPSTE24 in the clearance of clogged translocons relies on certain features of the protease that might be separable from those required for prelamin A cleavage. As shown previously (Ast et al., 2016), wild-type ZMPSTE24, but not catalytically dead versions, can efficiently replace yeast Ste24 when challenged with a clogging-prone substrate (Fig. 6). Interestingly, we also show here that some mutants, including Y399C and L462R, show poor prelamin A processing (∼25% and 6% of wild type, respectively), yet are largely proficient in the unclogging process. It is perhaps not surprising that the two different types of substrates (prelamin A and clogged proteins) might be handled differently. For instance, certain ZMPSTE24 mutations might prevent putative interactions between the protease and the translocon machinery or differ in their ability to permit access of the two different types of substrates to the active site. We speculate that like the Sec61 translocon itself, which is known to open laterally to release transmembrane spans (Gogala et al., 2014; Li et al., 2016; Pfeffer et al., 2015), ZMPSTE24 might also have the capacity to do so to facilitate transfer of a clogged substrate from the translocon pore to the ZMPSTE24 protease catalytic site. Isolating mutants specific to each function could help reveal important aspects about these processes. Thus far, we have not identified ZMPSTE24 mutants that are specifically deficient for de-clogging, but such mutants could be sought using our system and will provide further evidence that these activities are separable.

### Utility of our humanized yeast system for structure-function analysis of ZMPSTE24

**Fig. 7.**
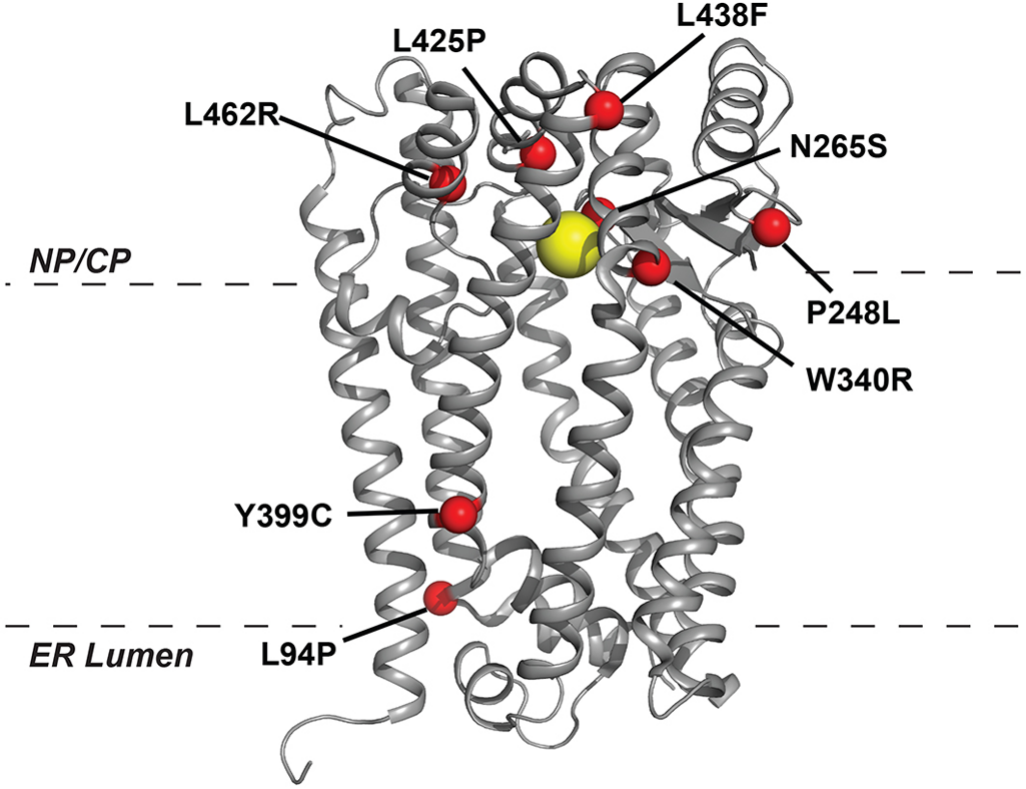
**Location of missense disease alleles in the ZMPSTE24 structure.** Positions of the missense disease alleles listed in Table 1 are indicated on a ribbon diagram of the ZMPSTE24 structure (PDB entry 2YPT; Quigley et al., 2013). The yellow ball represents the zinc ion at the catalytic site. Dashed lines indicate the approximate delineation of the lipid bilayer; the ER lumen and nucleoplasm/cytoplasm (NP/CP) are indicated.

In addition to its importance for understanding progeroid diseases and physiological aging, ZMPSTE24 is an intriguing molecule in terms of basic membrane protein biology. Although ZMPSTE24 has a canonical HEXXH zinc-binding motif that can coordinate zinc and mediates catalysis, the ZMPST24 structure is fundamentally different from other proteases, because catalysis occurs within an unusual enclosed intramembrane chamber (Clark et al., 2017; Pryor et al., 2013; Quigley et al., 2013). This novel structure elicits a number of questions concerning ZMPSTE24 enzyme mechanism, prelamin A access and positioning, and why prelamin A is the sole specific substrate known for ZMPSTE24. As a starting point, we focused here on the eight known ZMPSTE24 missense disease alleles. Six of the eight ZMPSTE24 disease alleles lie in residues highly conserved in ZMPSTE24/Ste24 among diverse species (the exceptions are L94 and Y399) and they cluster in two regions of the ZMPSTE24 structure (Fig. 7). Most mutations are at the top of the chamber, near the HEXXH catalytic motif, which coordinates the zinc ion (yellow). It has been suggested that these mutations could affect catalysis directly (N265S), impede substrate binding (L438F and L462R) or disrupt substrate entry into the chamber (P248L and W430R) (Quigley et al., 2013). Notably, two mutations L94P and Y399C map to the bottom side of the chamber, suggesting a functionally important activity in that region of ZMPSTE24, possibly in farnesyl binding for proper substrate positioning. As discussed above, an important finding here is that some disease mutations mainly produce an effect by destabilizing the protein, rather than affecting its enzymatic function *per se*.

In the long term, we expect that the humanized yeast system reported here, along with high-throughput mutagenesis, will allow us to answer mechanistic questions about how ZMPSTE24 functions and which features of misfolded versions of ZMPSTE24 recruit the UPS-dependent protein quality control machinery, an issue not well understood for any multispanning membrane protein. Our humanized yeast assay will also facilitate dissection of the prelamin A substrate to define a ZMPSTE24 consensus cleavage sequence and to probe the role of farnesyl for ZMPSTE24-mediated cleavage. Ultimately, the capacity to perform deep mutational scanning followed by specific screens and selections in yeast (Fowler et al., 2014; Starita and Fields, 2015) will facilitate isolation of separation-of-function alleles, in which ZMPSTE24 residues specific for prelamin A processing, declogging and antiviral activity can be identified.

## MATERIALS AND METHODS

### Plasmids and strains used in this study

Plasmids used in this study are listed in Table 3. All plasmids were constructed using standard molecular biology techniques, including NEBuilder^®^ HiFi Assembly (New England Biolabs, Ipswich, MA) and QuikChange^TM^ mutagenesis (Stratagene, San Diego, CA). When mutating *ZMPSTE24*-containing plasmids, *Escherichia coli* competent cells (Stbl2; Invitrogen, Carlsbad, CA) were transformed and grown at 30°C. ZMPSTE24 plasmids are *CEN*/*URA3* containing N-terminally *His_10_HA_3_*-tagged human *ZMPSTE24* expressed from the *PGK1* promoter. For ZMPSTE24-GFP plasmids, a PCR product encoding *GFP* was inserted in between codons 469 and 470 of the *ZMPSTE24* open reading frame, just subterminal to the C-terminal ER retrieval signal (Barrowman et al., 2008). Plasmid pSM3094 was constructed by subcloning a *Sac*II-­*Xho*I fragment containing N-terminally *His_10_HA_3_*-tagged yeast *STE24* from pSM1282 (Tam et al., 2001) into the same sites of pRS316. Plasmid pSM3283 is *CEN/HIS3* containing a single Flag epitope at the N terminus of ZMPSTE24 expressed from the *PGK1* promoter. Plasmid pSM3204 (expressing mCherry-Scs2_TM_) was constructed by NEBuilder^®^ HiFi Assembly of a PCR product from Kp173 (a generous gift of Rong Li, JHU School of Medicine, Baltimore, MD) into pRS315 (*CEN/LEU2*).

**Table 3.**
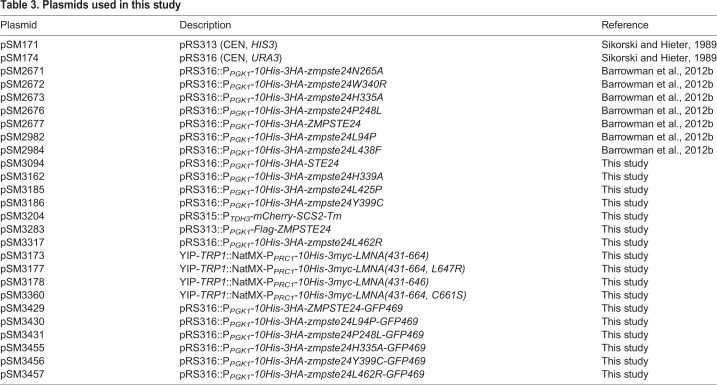
**Plasmids used in this study**

Plasmid pSM3173 is an integrating vector derived from Kp173. Briefly, PCR-generated fragments from the *PRC1* promoter (−800 to −1), *His_10_-myc_3_* and human *LMNA* (corresponding to amino acids 431-664) were recombined *in vitro* with a PCR-generated gapped Kp173 using NEBuilder^®^ HiFi Assembly. Plasmids pSM3177 (L647R) and pSM3360 (C661S) were generated with mutagenic oligonucleotides and QuikChange^TM^ mutagenesis using pSM3173 as template. Plasmid pSM3178, which expresses mature lamin A, was constructed by placing a stop codon after amino acid Y646 using NEBuilder^®^ HiFi Assembly. All manipulations with *LMNA* sequences used DH5α or NEB5 cells (New England Biolabs) for propagation. All integrating vectors in this study recombine at the *TRP1* locus by selecting with a nourseothricin/nourseothricin *N*-acetyl transferase system. Plasmid sequences and maps available upon request.

The yeast strains used in this study are listed in Table 4. To integrate *LMNA* constructs, integrating plasmids were linearized by *Eco*RV digestion and transformed into *ste24*Δ (SM4826) cells by standard yeast lithium acetate protocols. Transformants were selected on yeast extract peptone dextrose (YPD) containing 100 µg/ml nourseothricin. To generate the double mutants *ste24*Δ*doa10*Δ and *ste24*Δ*ste14*Δ, diploids were made by crossing single-mutant strains of opposite mating types and the double mutants were identified following sporulation and tetrad dissection. Strain SM6117 (*ste24*Δ *P_GAL1_-PDI1-DHFR-Nglyc-3HA*, clogger) was used previously (Ast et al., 2016). ZMPSTE24-expressing plasmids were transformed into strains and selected on minimal SC-Ura or SC-Ura-His plates.

**Table 4.**
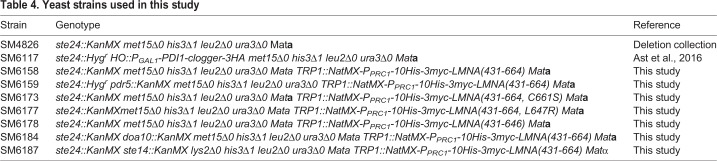
**Yeast strains used in this study**

### Yeast prelamin A cleavage assay

Typically, strains grown overnight in minimal medium (0.67% yeast nitrogen base, 0.5% ammonium sulfate, 2% glucose, supplemented with appropriate amino acids and supplements) were back-diluted in fresh medium for 4-6 h. Cells (1.5-2 OD_600_ cell equivalents) were pelleted, washed in water and lysed using NaOH pre-treatment and SDS protein sample buffer (Kushnirov, 2000) at 65°C for 10-15 min. For analysis, lysates were centrifuged at 21,000 ***g*** for 2 min and the supernatants (0.3 OD_600_ cell equivalents per lane) resolved on 10% SDS polyacrylamide gels. Proteins were transferred to nitrocellulose (Bio-Rad Trans-Blot^®^ Turbo^TM^) and the membrane blocked using Western Blocking Reagent (Roche). Lamin proteins were detected using mouse anti-myc antibodies (clone 4A6, Millipore cat #05-724; 1:10,000 dilution) decorated with goat anti-mouse secondary IRDye 680RD antibodies (LI-COR). Blots were re-probed using rat anti-HA (clone 3F10, Roche cat #11867423001; 1:10,000 dilution) to detect ZMPSTE24 and rabbit anti-Sec61 (1:10,000 dilution) as a loading control (a generous gift of Dr Randy Schekman, UC, Berkeley, CA), and visualized using goat anti-rat IRDye 680RD and goat anti-rabbit IRDye 800CW secondary antibodies (LI-COR). Prelamin A cleavage was calculated using ImageStudio Lite (LI-COR) by quantifying mature lamin A signal compared with total lamin A signal (prelamin A plus mature lamin A). ZMPSTE24 protein levels were quantified by measuring the HA signal in the entire region that contains both ZMPSTE24 bands and the intervening smear and normalizing this signal to the Sec61 loading control signal. Statistical analyses were performed by unpaired, two-tailed *t*-test (using Microsoft Excel) with *P*<0.05 considered to be significant.

### Clogger assay

Translocon clogging was examined essentially as previously described (Ast et al., 2016). Strain SM6117 transformed with vector or ZMPSTE24-expressing plasmid was grown overnight in SC-Ura with 2% sucrose as the carbon source. Strains were back-diluted in the same medium for 3 h and then induced by adding galactose to 2.5% for 6 h before collecting cells. SDS-PAGE, western transfer and blocking were performed as described for prelamin A cleavage. Clogger protein was detected using rat anti-HA (3F10, Roche) and LI-COR secondary antibodies. Inserted and clogged/cytoplasmic forms were quantified using ImageStudio Lite (LI-COR).

### Proteasome inhibition

To test the effect of proteasome inhibition on ZMPSTE24 protein levels and prelamin A cleavage, strain SM6159 (*ste24*Δ*pdr5*Δ *P_PRC1_-10His-3myc-LMNA_CT_*) transformed with the indicated ZMPSTE24 alleles was grown to log phase in SC-Ura medium. Transformed strains were then treated with either DMSO or 20 µM bortezomib (from 30 mM stock in DMSO, a generous gift from Peter Espenshade, JHU School of Medicine, Baltimore, MD) for 4 h at 30°C before lysate preparation, SDS-PAGE and western blotting, as described earlier. The proteasome inhibitor MG-132 was also tested with similar results (data not shown). The *pdr5*Δ mutation was introduced to enhance the efficacy of drug treatment, as previously described (Collins et al., 2010; Sung et al., 2016).

## Supplementary Material

Supplementary information

## References

[DMM033670C1] Agarwal A. K., Fryns J. P., Auchus R. J. and Garg A. (2003). Zinc metalloproteinase, ZMPSTE24, is mutated in mandibuloacral dysplasia. *Hum. Mol. Genet.* 12, 1995-2001. 10.1093/hmg/ddg213 12913070

[DMM033670C2] Agarwal A. K., Zhou X. J., Hall R. K., Nicholls K., Bankier A., Van Esch H., Fryns J.-P. and Garg A. (2006). Focal segmental glomerulosclerosis in patients with mandibuloacral dysplasia owing to ZMPSTE24 deficiency. *J. Investig. Med.* 54, 208-213. 10.2310/6650.2006.05068 17152860

[DMM033670C3] Ahmad Z., Zackai E., Medne L. and Garg A. (2010). Early onset mandibuloacral dysplasia due to compound heterozygous mutations in ZMPSTE24. *Am. J. Med. Genet. A* 152A, 2703-2710. 10.1002/ajmg.a.33664 20814950 PMC2965306

[DMM033670C4] Akinci B., Sankella S., Gilpin C., Ozono K., Garg A. and Agarwal A. K. (2017). Progeroid syndrome patients with ZMPSTE24 deficiency could benefit when treated with rapamycin and dimethylsulfoxide. *Cold Spring Harb. Mol. Case Stud.* 3, a001339 10.1101/mcs.a001339 28050601 PMC5171694

[DMM033670C5] Amaral M. D. (2015). Novel personalized therapies for cystic fibrosis: treating the basic defect in all patients. *J. Intern. Med.* 277, 155-166. 10.1111/joim.12314 25266997

[DMM033670C6] Ast T., Michaelis S. and Schuldiner M. (2016). The protease Ste24 clears clogged translocons. *Cell* 164, 103-114. 10.1016/j.cell.2015.11.053 26771486 PMC4715265

[DMM033670C7] Barrowman J. and Michaelis S. (2009). ZMPSTE24, an integral membrane zinc metalloprotease with a connection to progeroid disorders. *Biol. Chem.* 390, 761-773. 10.1515/BC.2009.080 19453269

[DMM033670C8] Barrowman J. and Michaelis S. (2011). CAAX processing and yeast a-factor biogenesis. *Enzymes, Vol 30: Protein Prenylation, Pt B* 30, 13-41.

[DMM033670C9] Barrowman J., Hamblet C., George C. M. and Michaelis S. (2008). Analysis of prelamin A biogenesis reveals the nucleus to be a CaaX processing compartment. *Mol. Biol. Cell* 19, 5398-5408. 10.1091/mbc.e08-07-0704 18923140 PMC2592638

[DMM033670C10] Barrowman J., Hamblet C., Kane M. S. and Michaelis S. (2012a). Requirements for efficient proteolytic cleavage of prelamin A by ZMPSTE24. *PLoS ONE* 7, e32120 10.1371/journal.pone.0032120 22355414 PMC3280227

[DMM033670C11] Barrowman J., Wiley P. A., Hudon-Miller S. E., Hrycyna C. A. and Michaelis S. (2012b). Human ZMPSTE24 disease mutations: residual proteolytic activity correlates with disease severity. *Hum. Mol. Genet.* 21, 4084-4093. 10.1093/hmg/dds233 22718200 PMC3428156

[DMM033670C12] Beck L. A., Hosick T. J. and Sinensky M. (1990). Isoprenylation is required for the processing of the lamin A precursor. *J. Cell Biol.* 110, 1489-1499. 10.1083/jcb.110.5.1489 2335559 PMC2200179

[DMM033670C13] Ben Yaou R., Navarro C., Quijano-Roy S., Bertrand A. T., Massart C., De Sandre-Giovannoli A., Cadinanos J., Mamchaoui K., Butler-Browne G., Estournet B. et al. (2011). Type B mandibuloacral dysplasia with congenital myopathy due to homozygous ZMPSTE24 missense mutation. *Eur. J. Hum. Genet.* 19, 647-654. 10.1038/ejhg.2010.256 21267004 PMC3110044

[DMM033670C14] Bergo M. O., Gavino B., Ross J., Schmidt W. K., Hong C., Kendall L. V., Mohr A., Meta M., Genant H., Jiang Y. et al. (2002). Zmpste24 deficiency in mice causes spontaneous bone fractures, muscle weakness, and a prelamin A processing defect. *Proc. Natl. Acad. Sci. USA* 99, 13049-13054. 10.1073/pnas.192460799 12235369 PMC130584

[DMM033670C15] Boyartchuk V. L. and Rine J. (1998). Roles of prenyl protein proteases in maturation of Saccharomyces cerevisiae a-factor. *Genetics* 150, 95-101. 9725832 10.1093/genetics/150.1.95 PMC1460331

[DMM033670C16] Brady G. F., Kwan R., Ulintz P. J., Nguyen P., Bassirian S., Basrur V., Nesvizhskii A. I., Loomba R. and Omary M. B. (2018). Nuclear lamina genetic variants, including a truncated LAP2, in twins and siblings with nonalcoholic fatty liver disease. *Hepatology* 67, 1710-1725. 10.1002/hep.29522 28902428 PMC5849478

[DMM033670C17] Brodsky J. L. (2012). Cleaning up: ER-associated degradation to the rescue. *Cell* 151, 1163-1167. 10.1016/j.cell.2012.11.012 23217703 PMC3521611

[DMM033670C18] Butin-Israeli V., Adam S. A., Goldman A. E. and Goldman R. D. (2012). Nuclear lamin functions and disease. *Trends Genet.* 28, 464-471. 10.1016/j.tig.2012.06.001 22795640 PMC3633455

[DMM033670C19] Capell B. C. and Collins F. S. (2006). Human laminopathies: nuclei gone genetically awry. *Nat. Rev. Genet.* 7, 940-952. 10.1038/nrg1906 17139325

[DMM033670C20] Clark K. M., Jenkins J. L., Fedoriw N. and Dumont M. E. (2017). Human CaaX protease ZMPSTE24 expressed in yeast: Structure and inhibition by HIV protease inhibitors. *Protein Sci.* 26, 242-257. 10.1002/pro.3074 27774687 PMC5275749

[DMM033670C21] Coffinier C., Hudon S. E., Farber E. A., Chang S. Y., Hrycyna C. A., Young S. G. and Fong L. G. (2007). HIV protease inhibitors block the zinc metalloproteinase ZMPSTE24 and lead to an accumulation of prelamin A in cells. *Proc. Natl. Acad. Sci. USA* 104, 13432-13437. 10.1073/pnas.0704212104 17652517 PMC1948915

[DMM033670C22] Collins G. A., Gomez T. A., Deshaies R. J. and Tansey W. P. (2010). Combined chemical and genetic approach to inhibit proteolysis by the proteasome. *Yeast* 27, 965-974. 10.1002/yea.1805 20625982 PMC3566228

[DMM033670C23] Cunningham V. J., D'Apice M. R., Licata N., Novelli G. and Cundy T. (2010). Skeletal phenotype of mandibuloacral dysplasia associated with mutations in ZMPSTE24. *Bone* 47, 591-597. 10.1016/j.bone.2010.06.004 20550970

[DMM033670C24] Davies B. S. J., Fong L. G., Yang S. H., Coffinier C. and Young S. G. (2009). The posttranslational processing of prelamin A and disease. *Annu. Rev. Genomics Hum. Genet.* 10, 153-174. 10.1146/annurev-genom-082908-150150 19453251 PMC2846822

[DMM033670C25] De Boeck K. and Amaral M. D. (2016). Progress in therapies for cystic fibrosis. *Lancet Respir Med.* 4, 662-674. 10.1016/S2213-2600(16)00023-0 27053340

[DMM033670C26] Dechat T., Adam S. A., Taimen P., Shimi T. and Goldman R. D. (2010). Nuclear lamins. *Cold Spring Harb. Perspect Biol.* 2, a000547 10.1101/cshperspect.a000547 20826548 PMC2964183

[DMM033670C27] Deng M. and Hochstrasser M. (2006). Spatially regulated ubiquitin ligation by an ER/nuclear membrane ligase. *Nature* 443, 827-831. 10.1038/nature05170 17051211

[DMM033670C28] De Sandre-Giovannoli A., Bernard R., Cau P., Navarro C., Amiel J., Boccaccio I., Lyonnet S., Stewart C. L., Munnich A., Le Merrer M. et al. (2003). Lamin a truncation in Hutchinson-Gilford progeria. *Science* 300, 2055 10.1126/science.1084125 12702809

[DMM033670C29] Dittmer T. A. and Misteli T. (2011). The lamin protein family. *Genome Biol.* 12, 222 10.1186/gb-2011-12-5-222 21639948 PMC3219962

[DMM033670C30] Dorado B. and Andrés V. (2017). A-type lamins and cardiovascular disease in premature aging syndromes. *Curr. Opin. Cell Biol.* 46, 17-25. 10.1016/j.ceb.2016.12.005 28086161

[DMM033670C31] Dutour A., Roll P., Gaborit B., Courrier S., Alessi M.-C., Tregouet D.-A., Angelis F., Robaglia-Schlupp A., Lesavre N., Cau P. et al. (2011). High prevalence of laminopathies among patients with metabolic syndrome. *Hum. Mol. Genet.* 20, 3779-3786. 10.1093/hmg/ddr294 21724554

[DMM033670C32] Eriksson M., Brown W. T., Gordon L. B., Glynn M. W., Singer J., Scott L., Erdos M. R., Robbins C. M., Moses T. Y., Berglund P. et al. (2003). Recurrent de novo point mutations in lamin A cause Hutchinson-Gilford progeria syndrome. *Nature* 423, 293-298. 10.1038/nature01629 12714972 PMC10540076

[DMM033670C33] Fowler D. M., Stephany J. J. and Fields S. (2014). Measuring the activity of protein variants on a large scale using deep mutational scanning. *Nat. Protoc.* 9, 2267-2284. 10.1038/nprot.2014.153 25167058 PMC4412028

[DMM033670C34] Fu B., Wang L., Li S. and Dorf M. E. (2017). ZMPSTE24 defends against influenza and other pathogenic viruses. *J. Exp. Med.* 214, 919-929. 10.1084/jem.20161270 28246125 PMC5379977

[DMM033670C35] Fujimura-Kamada K., Nouvet F. J. and Michaelis S. (1997). A novel membrane-associated metalloprotease, Ste24p, is required for the first step of NH2-terminal processing of the yeast a-factor precursor. *J. Cell Biol.* 136, 271-285. 10.1083/jcb.136.2.271 9015299 PMC2134828

[DMM033670C36] Galant D., Gaborit B., Desgrouas C., Abdesselam I., Bernard M., Levy N., Merono F., Coirault C., Roll P., Lagarde A. et al. (2016). A heterozygous ZMPSTE24 mutation associated with severe metabolic syndrome, ectopic fat accumulation, and dilated cardiomyopathy. *Cells* 5, 21 10.3390/cells5020021 PMC4931670 27120622

[DMM033670C37] Gardner R. G., Nelson Z. W. and Gottschling D. E. (2005). Degradation-mediated protein quality control in the nucleus. *Cell* 120, 803-815. 10.1016/j.cell.2005.01.016 15797381

[DMM033670C38] Gerace L. and Huber M. D. (2012). Nuclear lamina at the crossroads of the cytoplasm and nucleus. *J. Struct. Biol.* 177, 24-31. 10.1016/j.jsb.2011.11.007 22126840 PMC3261324

[DMM033670C39] Gogala M., Becker T., Beatrix B., Armache J.-P., Barrio-Garcia C., Berninghausen O. and Beckmann R. (2014). Structures of the Sec61 complex engaged in nascent peptide translocation or membrane insertion. *Nature* 506, 107-110. 10.1038/nature12950 24499919

[DMM033670C40] Gordon L. B., Rothman F. G., López-Otín C. and Misteli T. (2014). Progeria: a paradigm for translational medicine. *Cell* 156, 400-407. 10.1016/j.cell.2013.12.028 24485450 PMC6318797

[DMM033670C41] Gruenbaum Y. and Foisner R. (2015). Lamins: nuclear intermediate filament proteins with fundamental functions in nuclear mechanics and genome regulation. *Annu. Rev. Biochem.* 84, 131-164. 10.1146/annurev-biochem-060614-034115 25747401

[DMM033670C42] Guerriero C. J. and Brodsky J. L. (2012). The delicate balance between secreted protein folding and endoplasmic reticulum-associated degradation in human physiology. *Physiol. Rev.* 92, 537-576. 10.1152/physrev.00027.2011 22535891 PMC4162396

[DMM033670C43] Harhouri K., Navarro C., Baquerre C., Da Silva N., Bartoli C., Casey F., Mawuse G. K., Doubaj Y., Levy N. and De Sandre-Giovannoli A. (2016). Antisense-based progerin downregulation in hgps-like patients' cells. *Cells* 5, 31 10.3390/cells5030031 PMC5040973 27409638

[DMM033670C44] Haye D., Dridi H., Levy J., Lambert V., Lambert M., Agha M., Adjimi F., Kohlhase J., Lipsker D. and Verloes A. (2016). Failure of ossification of the occipital bone in mandibuloacral dysplasia type B. *Am. J. Med. Genet. A* 170, 2750-2755. 10.1002/ajmg.a.37825 27410998

[DMM033670C45] Hennekes H. and Nigg E. A. (1994). The role of isoprenylation in membrane attachment of nuclear lamins. A single point mutation prevents proteolytic cleavage of the lamin A precursor and confers membrane binding properties. *J. Cell Sci.* 107, 1019-1029. 8056827 10.1242/jcs.107.4.1019

[DMM033670C46] Huyer G., Piluek W. F., Fansler Z., Kreft S. G., Hochstrasser M., Brodsky J. L. and Michaelis S. (2004). Distinct machinery is required in Saccharomyces cerevisiae for the endoplasmic reticulum-associated degradation of a multispanning membrane protein and a soluble luminal protein. *J. Biol. Chem.* 279, 38369-38378. 10.1074/jbc.M402468200 15252059

[DMM033670C47] Kayatekin C., Amasino A., Gaglia G., Flannick J., Bonner J. M., Fanning S., Narayan P., Barrasa M. I., Pincus D., Landgraf D. et al. (2018). Translocon declogger Ste24 protects against IAPP oligomer-induced proteotoxicity. *Cell* 173, 62-73 e9. 10.1016/j.cell.2018.02.026 29526462 PMC5945206

[DMM033670C48] Kushnirov V. V. (2000). Rapid and reliable protein extraction from yeast. *Yeast* 16, 857-860. 10.1002/1097-0061(20000630)16:9<857::AID-YEA561>3.0.CO;2-B 10861908

[DMM033670C49] Li L., Park E., Ling J. J., Ingram J., Ploegh H. and Rapoport T. A. (2016). Crystal structure of a substrate-engaged SecY protein-translocation channel. *Nature* 531, 395-399. 10.1038/nature17163 26950603 PMC4855518

[DMM033670C50] Mallampalli M. P., Huyer G., Bendale P., Gelb M. H. and Michaelis S. (2005). Inhibiting farnesylation reverses the nuclear morphology defect in a HeLa cell model for Hutchinson-Gilford progeria syndrome. *Proc. Natl. Acad. Sci. USA* 102, 14416-14421. 10.1073/pnas.0503712102 16186497 PMC1242289

[DMM033670C51] Mattout A., Dechat T., Adam S. A., Goldman R. D. and Gruenbaum Y. (2006). Nuclear lamins, diseases and aging. *Curr. Opin. Cell Biol.* 18, 335-341. 10.1016/j.ceb.2006.03.007 16632339

[DMM033670C52] Mehmood S., Marcoux J., Gault J., Quigley A., Michaelis S., Young S. G., Carpenter E. P. and Robinson C. V. (2016). Mass spectrometry captures off-target drug binding and provides mechanistic insights into the human metalloprotease ZMPSTE24. *Nat. Chem.* 8, 1152-1158. 10.1038/nchem.2591 27874871 PMC5123592

[DMM033670C53] Merideth M. A., Gordon L. B., Clauss S., Sachdev V., Smith A. C. M., Perry M. B., Brewer C. C., Zalewski C., Kim H. J., Solomon B. et al. (2008). Phenotype and course of Hutchinson-Gilford progeria syndrome. *N. Engl. J. Med.* 358, 592-604. 10.1056/NEJMoa0706898 18256394 PMC2940940

[DMM033670C54] Michaelis S. and Barrowman J. (2012). Biogenesis of the Saccharomyces cerevisiae pheromone a-factor, from yeast mating to human disease. *Microbiol. Mol. Biol. Rev.* 76, 626-651. 10.1128/MMBR.00010-12 22933563 PMC3429625

[DMM033670C55] Michaelis S. and Hrycyna C. A. (2013). Biochemistry. A protease for the ages. *Science* 339, 1529-1530. 10.1126/science.1236764 23539586

[DMM033670C56] Miyoshi Y., Akagi M., Agarwal A. K., Namba N., Kato-Nishimura K., Mohri I., Yamagata M., Nakajima S., Mushiake S., Shima M. et al. (2008). Severe mandibuloacral dysplasia caused by novel compound heterozygous ZMPSTE24 mutations in two Japanese siblings. *Clin. Genet.* 73, 535-544. 10.1111/j.1399-0004.2008.00992.x 18435794 PMC2732118

[DMM033670C57] Moulson C. L., Go G., Gardner J. M., van der Wal A. C., Smitt J. H., van Hagen J. M. and Miner J. H. (2005). Homozygous and compound heterozygous mutations in ZMPSTE24 cause the laminopathy restrictive dermopathy. *J. Invest. Dermatol.* 125, 913-919. 10.1111/j.0022-202X.2005.23846.x 16297189 PMC1360172

[DMM033670C58] Navarro C. L., Cadiñanos J., De Sandre-Giovannoli A., Bernard R., Courrier S., Boccaccio I., Boyer A., Kleijer W. J., Wagner A., Giuliano F. et al. (2005). Loss of ZMPSTE24 (FACE-1) causes autosomal recessive restrictive dermopathy and accumulation of Lamin A precursors. *Hum. Mol. Genet.* 14, 1503-1513. 10.1093/hmg/ddi159 15843403

[DMM033670C59] Navarro C. L., Esteves-Vieira V., Courrier S., Boyer A., Duong Nguyen T., Huong le T. T., Meinke P., Schröder W., Cormier-Daire V., Sznajer Y. et al. (2014). New ZMPSTE24 (FACE1) mutations in patients affected with restrictive dermopathy or related progeroid syndromes and mutation update. *Eur. J. Hum. Genet.* 22, 1002-1011. 10.1038/ejhg.2013.258 24169522 PMC4350588

[DMM033670C60] Newman M. J., Foster D. L., Wilson T. H. and Kaback H. R. (1981). Purification and reconstitution of functional lactose carrier from Escherichia coli. *J. Biol. Chem.* 256, 11804-11808. 7028742

[DMM033670C61] Pendas A. M., Zhou Z., Cadinanos J., Freije J. M., Wang J., Hultenby K., Astudillo A., Wernerson A., Rodriguez F., Tryggvason K. et al. (2002). Defective prelamin A processing and muscular and adipocyte alterations in Zmpste24 metalloproteinase-deficient mice. *Nat. Genet.* 31, 94-99. 10.1038/ng871 11923874

[DMM033670C62] Pfeffer S., Burbaum L., Unverdorben P., Pech M., Chen Y., Zimmermann R., Beckmann R. and Förster F. (2015). Structure of the native Sec61 protein-conducting channel. *Nat. Commun.* 6, 8403 10.1038/ncomms9403 26411746 PMC4598622

[DMM033670C63] Pryor E. E. Jr, Horanyi P. S., Clark K. M., Fedoriw N., Connelly S. M., Koszelak-Rosenblum M., Zhu G., Malkowski M. G., Wiener M. C. and Dumont M. E. (2013). Structure of the integral membrane protein CAAX protease Ste24p. *Science* 339, 1600-1604. 10.1126/science.1232048 23539602 PMC4136949

[DMM033670C64] Quigley A., Dong Y. Y., Pike A. C. W., Dong L., Shrestha L., Berridge G., Stansfeld P. J., Sansom M. S., Edwards A. M., Bountra C. et al. (2013). The structural basis of ZMPSTE24-dependent laminopathies. *Science* 339, 1604-1607. 10.1126/science.1231513 23539603

[DMM033670C65] Ragnauth C. D., Warren D. T., Liu Y., McNair R., Tajsic T., Figg N., Shroff R., Skepper J. and Shanahan C. M. (2010). Prelamin A acts to accelerate smooth muscle cell senescence and is a novel biomarker of human vascular aging. *Circulation* 121, 2200-2210. 10.1161/CIRCULATIONAHA.109.902056 20458013

[DMM033670C66] Rath A., Glibowicka M., Nadeau V. G., Chen G. and Deber C. M. (2009). Detergent binding explains anomalous SDS-PAGE migration of membrane proteins. *Proc. Natl. Acad. Sci. USA* 106, 1760-1765. 10.1073/pnas.0813167106 19181854 PMC2644111

[DMM033670C67] Ravid T., Kreft S. G. and Hochstrasser M. (2006). Membrane and soluble substrates of the Doa10 ubiquitin ligase are degraded by distinct pathways. *EMBO J.* 25, 533-543. 10.1038/sj.emboj.7600946 16437165 PMC1383530

[DMM033670C68] Schmidt W. K., Tam A. and Michaelis S. (2000). Reconstitution of the Ste24p-dependent N-terminal proteolytic step in yeast a-factor biogenesis. *J. Biol. Chem.* 275, 6227-6233. 10.1074/jbc.275.9.6227 10692417

[DMM033670C69] Shackleton S., Smallwood D. T., Clayton P., Wilson L. C., Agarwal A. K., Garg A. and Trembath R. C. (2005). Compound heterozygous ZMPSTE24 mutations reduce prelamin A processing and result in a severe progeroid phenotype. *J. Med. Genet.* 42, e36 10.1136/jmg.2004.029751 15937076 PMC1736080

[DMM033670C70] Sikorski R. S. and Hieter P. (1989). A system of shuttle vectors and yeast host strains designed for efficient manipulation of DNA in Saccharomyces cerevisiae. *Genetics* 122, 19-27. 2659436 10.1093/genetics/122.1.19 PMC1203683

[DMM033670C71] Sinensky M., Fantle K., Trujillo M., McLain T., Kupfer A. and Dalton M. (1994). The processing pathway of prelamin A. *J. Cell Sci.* 107, 61-67. 8175923 10.1242/jcs.107.1.61

[DMM033670C72] Smigiel R., Jakubiak A., Esteves-Vieira V., Szela K., Halon A., Jurek T., Lévy N. and De Sandre-Giovannoli A. (2010). Novel frameshifting mutations of the ZMPSTE24 gene in two siblings affected with restrictive dermopathy and review of the mutations described in the literature. *Am. J. Med. Genet. A* 152A, 447-452. 10.1002/ajmg.a.33221 20101687

[DMM033670C73] Starita L. M. and Fields S. (2015). Deep mutational scanning: a highly parallel method to measure the effects of mutation on protein function. *Cold Spring Harb. Protoc.* 2015, 711-714. 10.1101/pdb.top077503 26240414

[DMM033670C74] Sung M.-K., Reitsma J. M., Sweredoski M. J., Hess S. and Deshaies R. J. (2016). Ribosomal proteins produced in excess are degraded by the ubiquitin-proteasome system. *Mol. Biol. Cell* 27, 2642-2652. 10.1091/mbc.e16-05-0290 27385339 PMC5007085

[DMM033670C75] Swanson R., Locher M. and Hochstrasser M. (2001). A conserved ubiquitin ligase of the nuclear envelope/endoplasmic reticulum that functions in both ER-associated and Matalpha2 repressor degradation. *Genes Dev.* 15, 2660-2674. 10.1101/gad.933301 11641273 PMC312819

[DMM033670C76] Tam A., Nouvet F. J., Fujimura-Kamada K., Slunt H., Sisodia S. S. and Michaelis S. (1998). Dual roles for Ste24p in yeast a-factor maturation: NH2-terminal proteolysis and COOH-terminal CAAX processing. *J. Cell Biol.* 142, 635-649. 10.1083/jcb.142.3.635 9700155 PMC2148179

[DMM033670C77] Tam A., Schmidt W. K. and Michaelis S. (2001). The multispanning membrane protein Ste24p catalyzes CAAX proteolysis and NH2-terminal processing of the yeast a-factor precursor. *J. Biol. Chem.* 276, 46798-46806. 10.1074/jbc.M106150200 11581258

[DMM033670C78] Thill M., Nguyen T. D., Wehnert M., Fischer D., Hausser I., Braun S. and Jackisch C. (2008). Restrictive dermopathy: a rare laminopathy. *Arch. Gynecol. Obstet.* 278, 201-208. 10.1007/s00404-008-0676-6 18470519

[DMM033670C79] Varela I., Pereira S., Ugalde A. P., Navarro C. L., Suárez M. F., Cau P., Cadinanos J., Osorio F. G., Foray N., Cobo J. et al. (2008). Combined treatment with statins and aminobisphosphonates extends longevity in a mouse model of human premature aging. *Nat. Med.* 14, 767-772. 10.1038/nm1786 18587406

[DMM033670C80] Wang M. and Casey P. J. (2016). Protein prenylation: unique fats make their mark on biology. *Nat. Rev. Mol. Cell Biol.* 17, 110-122. 10.1038/nrm.2015.11 26790532

[DMM033670C81] Wang Y., Lichter-Konecki U., Anyane-Yeboa K., Shaw J. E., Lu J. T., Östlund C., Shin J.-Y., Clark L. N., Gundersen G. G., Nagy P. L. et al. (2016). A mutation abolishing the ZMPSTE24 cleavage site in prelamin A causes a progeroid disorder. *J. Cell Sci.* 129, 1975-1980. 10.1242/jcs.187302 27034136 PMC4878994

[DMM033670C82] Worman H. J., Fong L. G., Muchir A. and Young S. G. (2009). Laminopathies and the long strange trip from basic cell biology to therapy. *J. Clin. Invest.* 119, 1825-1836. 10.1172/JCI37679 19587457 PMC2701866

[DMM033670C83] Yang S. H., Andres D. A., Spielmann H. P., Young S. G. and Fong L. G. (2008). Progerin elicits disease phenotypes of progeria in mice whether or not it is farnesylated. *J. Clin. Invest.* 118, 3291-3300. 10.1172/JCI35876 18769635 PMC2525700

[DMM033670C84] Young S. G., Fong L. G. and Michaelis S. (2005). Prelamin A, Zmpste24, misshapen cell nuclei, and progeria--new evidence suggesting that protein farnesylation could be important for disease pathogenesis. *J. Lipid Res.* 46, 2531-2558. 10.1194/jlr.R500011-JLR200 16207929

