# Supplementary material for: ZMPSTE24 missense mutations that cause progeroid diseases decrease prelamin A cleavage activity and/or protein stability
Source: Dis Model Mech. 2018 Jul 13;11(7):dmm033670. doi: 10.1242/dmm.033670 (PMC6078402; doi:10.1242/dmm.033670)
Supplement: Supplementary information [file dmm-11-033670-s1.pdf]

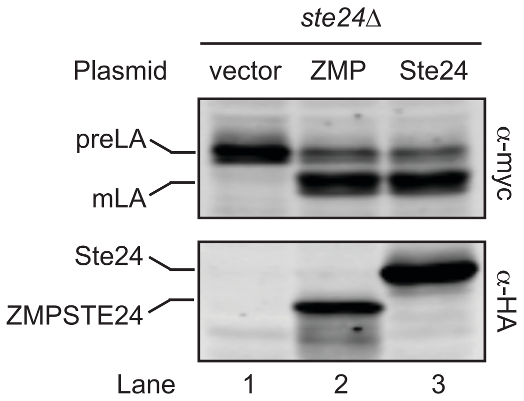

Figure S1. Yeast Ste24 can catalyze prelamin A processing as efficiently as human ZMPSTE24. Lysates were prepared from SM6158 (*ste24Δ myc-LMNA*$_{CT}$) cells transformed with vector only (Lane 1), pSM2677 (human *ZMPSTE24*, Lane 2) or pSM3094 (yeast *STE24*, Lane 3) and subjected to SDS-PAGE and Western blotting with α-myc antibodies to detect processing of prelamin A and α-HA antibodies to detect the ZMPSTE24 or Ste24 proteins, which both contain an N-terminal 10His-3HA epitope tag and are expressed from the same promoter. Although similar in size, ZMPSTE24 (475 amino acids) and Ste24 (453 amino acids) migrate significantly different by SDS-PAGE, with Ste24 surprisingly migrating more slowly than ZMPSTE24, despite its smaller size. However, anomalous SDS-PAGE migration patterns are not uncommon among membrane proteins, due to unpredictable differences in their detergent binding properties (Newman et al., 1981; Rath et al., 2009).

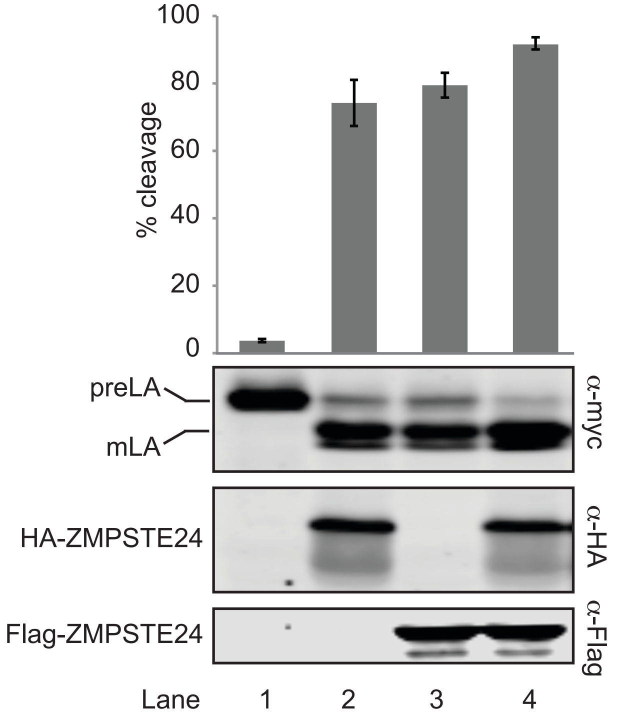

Figure S2. The extent of prelamin A processing depends on the amount of ZMPSTE24 cells express. Lysates were prepared from strain SM6158 (*ste24*Δ *myc-LMNA*$_{CT}$) containing empty vectors only (pRS313 and pRS316; Lane 1), HA-ZMPSTE24 and empty vector (pSM2677 and pRS313; Lane 2), Flag-ZMPSTE24 and empty vector (pSM3283 and pRS316; Lane 3) or HA-ZMPSTE24 and Flag-ZMPSTE24 (pSM2677 and pSM3283; Lane 4) and were analyzed by western blotting as described. Prelamin A cleavage is expressed as the mean ± s.d. from three independent trials. $p<0.05$ for vectors and one copy compared to two copies of ZMPSTE24. With a single copy of ZMPSTE24 expressed from the *PGK1* promoter, the prelamin A model substrate undergoes 65-80% processing (lanes 2 and 3), which increased to 90% with two copies (lane 4). It is possible that 100% processing cannot be achieved because a small percentage of prelamin A is not farnesylated.

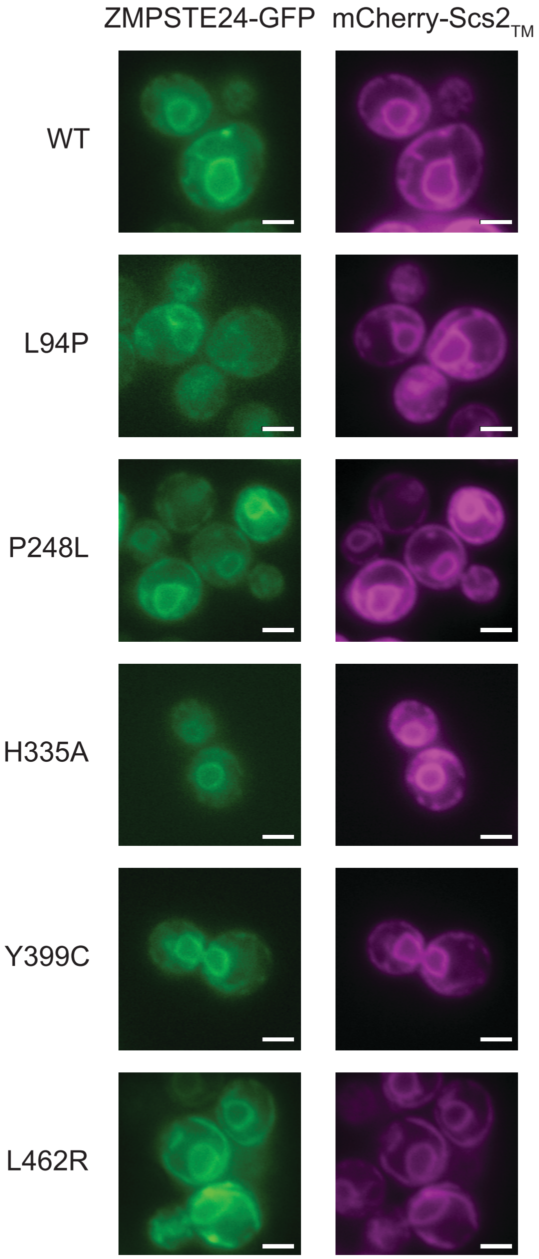

Figure S3. WT and ZMPSTE24-GFP variants localize to the perinuclear and cortical ER membranes. Strain SM4826 (*ste24Δ*) was transformed with mCherry-$Scs2_{TM}$ (pSM3204) and the indicated ZMPSTE24-GFP variants were visualized by fluorescence microscopy. mCherry-$Scs2_{TM}$ is an ER marker. Shown are the ZMPSTE24 mutants having the most severe prelamin defects. All HA-ZMPSTE24 mutants show no obvious localization changes by indirect immunofluorescence (data not shown). Scale bar, 2μM.

Disease Models & Mechanisms • Supplementary information

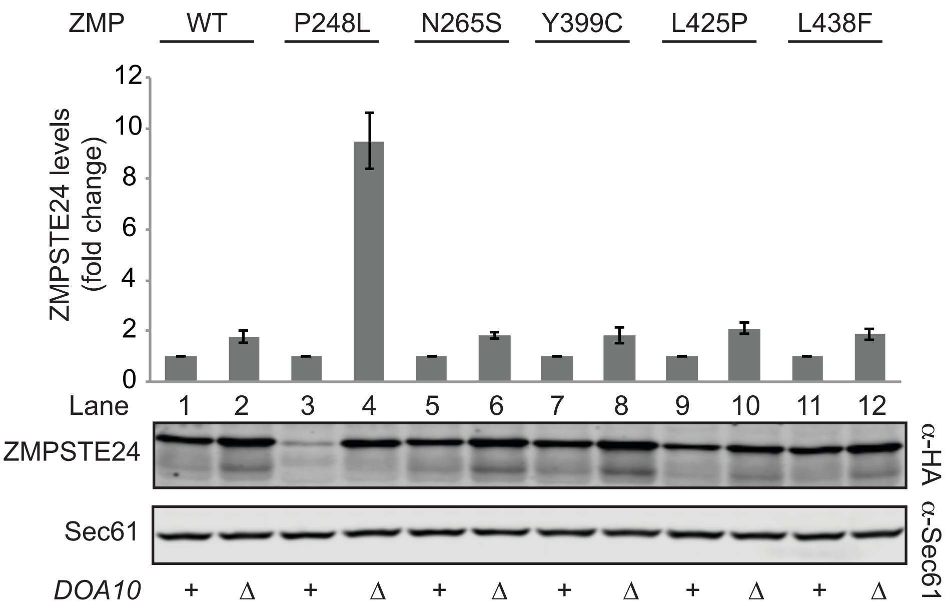

Figure S4. ZMPSTE24 protein levels are increased in the *doa10*Δ mutant.
Strains SM6158 (*ste24*Δ *myc-LMNA*$_{CT}$) or SM6184 (*ste24*Δ*doa10*Δ *myc-LMNA*$_{CT}$) expressing the indicated ZMPSTE24 variants were analyzed by SDS-PAGE and western blotting as described in Figure 5. ZMPSTE24 steady-state levels were normalized to the loading control Sec61, and the mean ± s.d. for three independent experiments is shown. $p<0.05$ for all ZMPSTE24 variants between WT and *doa10*Δ cells.

Disease Models & Mechanisms • Supplementary information

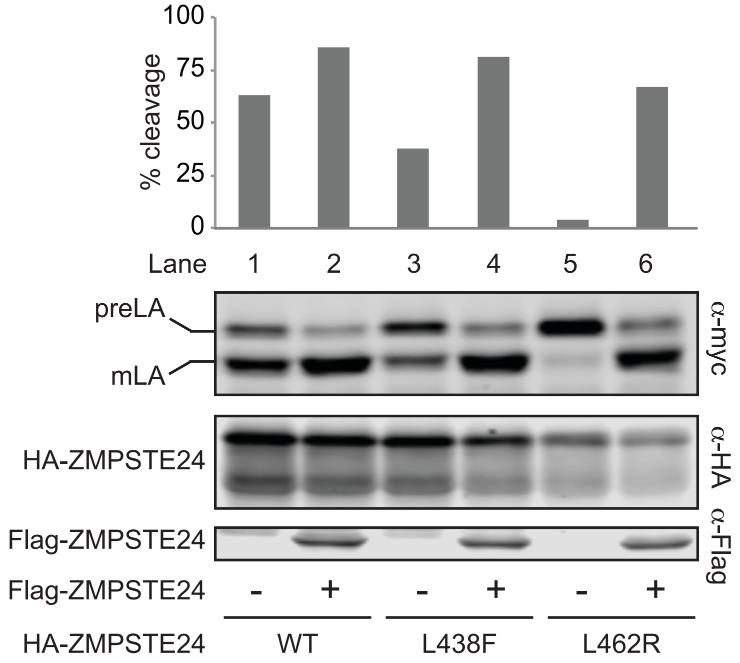

Figure S5. ZMPSTE24 disease alleles do not exhibit dominance in the yeast prelamin A cleavage assay. Strain SM6158 (*ste24Δ myc-LMNA*$_{CT}$) transformed with the indicated HA-ZMPSTE24 disease alleles and either pRS313 (vector only; -) or pSM3283 (which expresses WT Flag-ZMPSTE24; +) were assayed for in vivo prelamin A cleavage. Lysates were resolved by SDS-PAGE and proteins detected by immunoblotting against myc (prelamin A), HA (HA-ZMPSTE24) and Flag (Flag-ZMPSTE24). So long as at least one ZMPSTE24 allele is WT (lanes 2, 4 and 6) processing of prelamin A is close to 70%, even if a mutant allele is present.
